# The CLN3 gene and protein: What we know

**DOI:** 10.1002/mgg3.859

**Published:** 2019-09-30

**Authors:** Myriam Mirza, Anna Vainshtein, Alberto DiRonza, Uma Chandrachud, Luke J. Haslett, Michela Palmieri, Stephan Storch, Janos Groh, Niv Dobzinski, Gennaro Napolitano, Carolin Schmidtke, Danielle M. Kerkovich

**Affiliations:** ^1^ Mila’s Miracle Foundation Boulder Colorado; ^2^ Craft Science Inc. Thornhill Canada; ^3^ Baylor College of Medicine Houston Texas; ^4^ Jan and Dan Duncan Neurological Research Institute at Texas Children’s Hospital Houston Texas; ^5^ Center for Genomic Medicine Massachusetts General Hospital/Harvard Medical School Boston Massachusetts; ^6^ School of Biosciences Cardiff University Cardiff UK; ^7^ Biochemistry University Medical Center Hamburg‐Eppendorf Hamburg Germany; ^8^ Neurology University Hospital Wuerzburg Wuerzburg Germany; ^9^ Biochemistry and Biophysics UCSF School of Medicine San Francisco California; ^10^ Telethon Institute of Genetics and Medicine Napoli Italy; ^11^ Beyond Batten Disease Foundation Austin Texas

**Keywords:** Batten, CLN3, JNCL, juvenile Batten, neuronal ceroid lipofuscinosis

## Abstract

**Background:**

One of the most important steps taken by Beyond Batten Disease Foundation in our quest to cure juvenile Batten (CLN3) disease is to understand the State of the Science. We believe that a strong understanding of where we are in our experimental understanding of the CLN3 gene, its regulation, gene product, protein structure, tissue distribution, biomarker use, and pathological responses to its deficiency, lays the groundwork for determining therapeutic action plans.

**Objectives:**

To present an unbiased comprehensive reference tool of the experimental understanding of the CLN3 gene and gene product of the same name.

**Methods:**

BBDF compiled all of the available CLN3 gene and protein data from biological databases, repositories of federally and privately funded projects, patent and trademark offices, science and technology journals, industrial drug and pipeline reports as well as clinical trial reports and with painstaking precision, validated the information together with experts in Batten disease, lysosomal storage disease, lysosome/endosome biology.

**Results:**

The finished product is an indexed review of the CLN3 gene and protein which is not limited in page size or number of references, references all available primary experiments, and does not draw conclusions for the reader.

**Conclusions:**

Revisiting the experimental history of a target gene and its product ensures that inaccuracies and contradictions come to light, long‐held beliefs and assumptions continue to be challenged, and information that was previously deemed inconsequential gets a second look. Compiling the information into one manuscript with all appropriate primary references provides quick clues to which studies have been completed under which conditions and what information has been reported. This compendium does not seek to replace original articles or subtopic reviews but provides an historical roadmap to completed works.

## Graphical abstract

The CLN3 gene, its regulation, gene product, CLN3 protein structure, tissue distribution, biomarker use, and pathological responses to its deficiency.

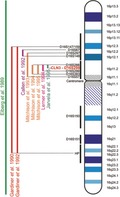

## 1 INTRODUCTION

Basic knowledge of the expression, regulation, structure, and function transmembrane‐bound and other proteins, enables the discovery of compounds to modulate their behavior. Along with analyses of disease‐causing mutations, investigators pursue creative approaches to restore protein function(s) and their associated pathways. Therefore, it is critically important that academicians, pharmaceutical investigators, and clinician scientists, be provided with a complete, easy‐to‐access, and an up‐to‐date State of the Science. Historically, one relied on review articles designed to summarize current thinking. However, an information explosion fueled by advances in molecular biology, genetic engineering, and new animal models, coupled with competing hypotheses of authors and size limits of review articles has resulted in the production of irregular, nonsystematic review articles in disease research leading to unintentional bias and widening knowledge gaps. To combat this problem, investigators new to the field must spend hundreds of hours sifting, reading, and evaluating original publications; defeating the purpose for which review articles were created.

Beyond Batten Disease Foundation (BBDF) has taken a lead to help support new and existing researchers in their quest for experimentally proven, unbiased, information. This manuscript is part of a larger strategic plan to advance research in Batten disease by fueling the creation of key physical and informational resources. The foundation worked with Thomson Reuters to gather referenced *CLN3* gene and CLN3 protein information and with painstaking attention to detail, validated the information together with experts in CLN3 disease, lysosomal storage disease, and lysosome/endosome biology. The resultant indexed review of *CLN3* and CLN3 is not limited in size, focuses on information from original articles, is reviewed by in‐area experts and inclusion in validated databases, and does not draw conclusions. By collecting all of the available information into a single, searchable reference manual, this review saves valuable time and ensures all topic areas are covered; however, readers will still need to review original literature cited here.

The information found within this reference tool is cultivated by: (a) MetaBase™ (version 6.20), a systems biology database, a former product of GeneGo, Integrity^SM^, (b) a drug and pipeline information database, Cortellis™, and (c) a drug and clinical trial information database, Thomson Innovation™, including patent information from around the world and public databases (December 2014 version), such as NCBI, Ensembl, dbSNP, UniProt, MGI and others. The information cultivated from these databases was then traced back its original source and rigorously reviewed. If the experiment was conducted more than once, all references were added.

The manual includes a research history of the *CLN3* gene, discussion of gene regulation, protein structure, tissue distribution, co‐regulated gene expression, biomarker use, and pathological responses to CLN3 protein deficiencies in yeast through humans. Supplementary materials include a list of CLN3 research tools and their associated first‐published reports.

The authors note that following data extraction from the information systems mentioned above, the information gathered here was verified and the associated primary literature was cited along with the experimental methods used. We believe bioinformatics databases such as those mentioned above offer scientists the opportunity to access and cross‐reference a wide variety of biologically relevant data providing new insights and further means to validate their discoveries. However, the authors would like to stress that the databases listed here were used only to provide a framework for the document. The rapid release of new data from various –omics and other programs annotated using computational analysis sometimes leads to misinformation, which we found to be the case for the *CLN3* gene and protein. Therefore, the authors worked to provide their readership with direct access to primary, experimentally proven data. Finally, the authors diligently tried to avoid summarizing the information herein in support of one hypothesis over another or drawing any conclusions for the reader. This comprehensive agnostic presentation of experimental findings is meant to complement and not overlap investigator‐driven primary and review literature.

## 2 GENERAL INFORMATION

### 2.1 Description of the *CLN3* gene

**Figure 1:**
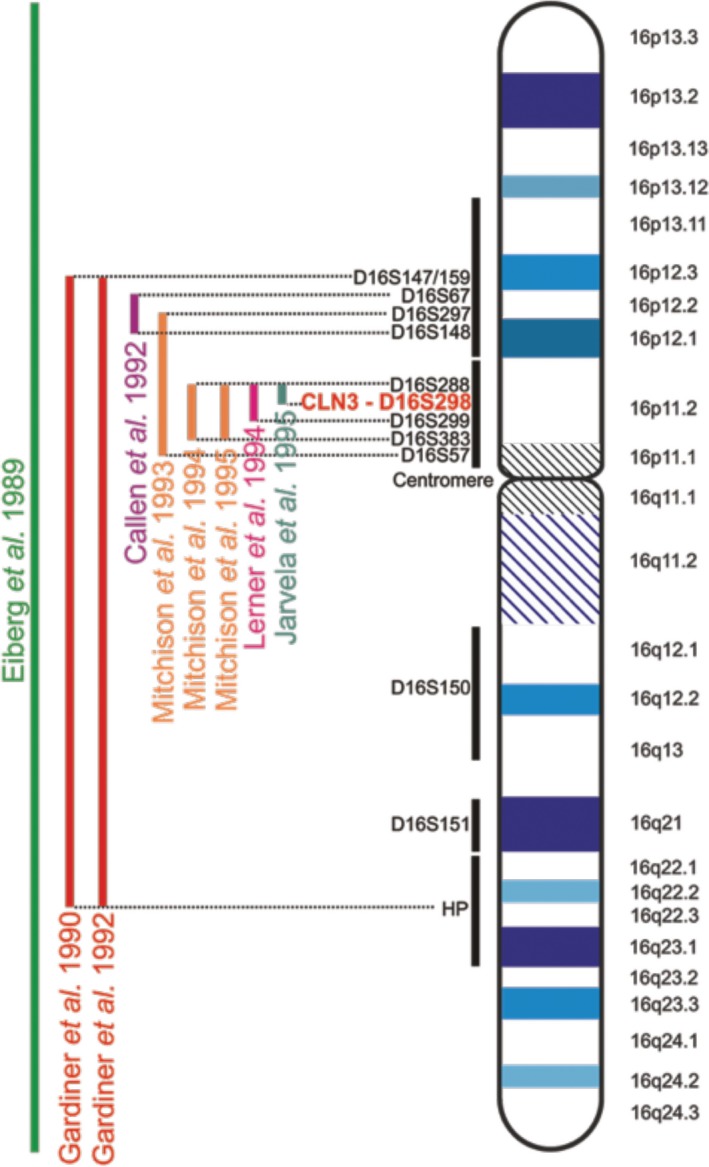
Linkage Mapping. The location of the gene responsible for JNCL was mapped to a region on chromosome 16. Initial findings placed the gene on the long arm of chromosome 16, due to its linkage with the haptoglobin (HP) locus. Later, the location of *CLN3* was narrowed down to markers tagging the 16p11.2 region. The dinucleotide marker D16S298 is located in an intron of the *CLN3* gene and thus represents the true location of *CLN3*

The official name of this gene is “ceroid‐lipofuscinosis, neuronal 3” and official symbol is *CLN3.* Less commonly used terms include BATTENIN, BTS, JNCL (Juvenile Neuronal Ceroid Lipofuscinosis), and MGC102840. *CLN3* was discovered using linkage analysis in search for the disease causing gene (mutation) in 48 children with progressive vision loss, seizures, decline of intellect and loss of motor ability (Eiberg, Gardiner, & Mohr, 1989). Researchers found a linkage between CLN3 disease and haptoglobin (140100) on chromosome 16q22 revealing the location of the CLN3 gene (Eiberg et al., 1989; Gardiner et al., 1990). Linkage studies in larger groups of families followed by physical mapping of markers by mouse/human hybrid cell analysis and fluorescence in situ hybridization refined the coordinates of *CLN3* to the interval between D16S288 and D16S298 (see Figure 1: LinkageMapping, (Callen et al., 1991, 1992; Järvelä, Mitchison, Callen, et al., 1995; Järvelä, Mitchison, O'rawe, et al., 1995; Lerner et al., 1994; Mitchison, O'Rawe, Lerner, et al., 1995; Mitchison, O’Rawe, Taschner, et al., 1995; Mitchison et al., 1994; Mitchison, Williams, et al., 1993; Mitchison, Thompson, et al., 1993) The final pieces of the puzzle were added by the International Batten Disease Consortium in 1995 (Batten Disease Consortium, 1995). Today, we know that the cytogenetic location of *CLN3* is on the short arm of chromosome 16 at position 12.1 at Genomic coordinates chr16:28,466,653–28,492,302 [OMIM 607042] (NCBI gene entry, 1,201 (Järvelä, Mitchison, O'rawe, et al., 1995; Järvelä, Mitchison, Callen, et al., 1995; Mitchison, O'Rawe, Lerner, et al., 1995; Mitchison, Williams, et al., 1993).

In 1997, Mitchison and colleagues reported the genomic structure and complete nucleotide sequence of *CLN3*, with an estimated number of 15 exons that span 15 kilobases (kb), (Mitchison et al., 1997). Sequence comparisons between *CLN3* and homologous expressed sequence tags suggest alternative splicing of the gene and at least 1 additional upstream exon. Marker loci in strong allelic association with the disease loci have been identified (Mole & Gardiner, 1991).

*Haplotype analysis identified a homozygous deletion mutation of 966 base pairs (bps) in 73% of 200 affected JNCL patients from 16 different countries* (Mitchison, O'Rawe, Lerner, et al., 1995; Mitchison, Thompson, et al., 1993)*. This deletion mutation was originally believed to stretch 1.02 kb* (Batten Disease Consortium, 1995)*. As a result, many reviews and primary articles refer to this deletion as the “1.02 kb” deletion. However, it was later confirmed that the common deletion spans 966 bps and is therefore more appropriately called the “1 kb” deletion. Cultured fibroblasts from a JNCL patient homozygous for the common 1 kb deletion have been shown to express a major transcript of 521 bps and a minor transcript of 408 bps* (Kitzmüller, Haines, Codlin, Cutler, & Mole, 2008)*. The major transcript contains exon 6 spliced to exon 9 and is thought to encode a truncated* CLN3 *protein containing the first 153 amino acids of* CLN3 *plus an additional 28 novel amino acids resulting from an out‐of‐frame RNA sequence at the novel splice site. This gives rise to the following mutant protein sequence:*

**Table:** 

*1*	*MGGCAGSRRRFSDSEGEETVPEPRLPLLDHQGAHWKNAVGFWLLGLCNNFSYVVMLSAA*	*60*
*61*	*DILSHKRTSGNQSHVDPGPTPIPHNSSSRFDCNSVSTAAVLLADILPTLVIKLLAPLGLH*	*120*
*121*	*LLPYSPRVLVSGICAAGSFVLVAFSHSVGTSLCAISCCSHLLRPRTLEGKKKQRAQPGSP*	*180*
*181*	*S*	*181*

*(Underlined letters indicate truncated first 153 AAs of* CLN3 *followed by 28 novel AAs due to a frameshift at the novel splice site). It is important to note that there is an S written in error at position 166 in some GenBank entries (GenBank: EF587245/1 and publications* (Kitzmüller et al., 2008)*. This should be a “T” as shown above in yellow highlight (GenBank accession no AF077964 and AF077968, which are consistent with genomic sequence NG 008654.2).*

### 2.2 *CLN3* gene details

The full name of the gene is ceroid‐lipofuscinosis, neuronal 3, also known by the following symbols (CLN3, BTS, and JNCL). Gene product names include Batten disease protein, Batten, and Battenin. CLN3 gene product deficiency results in a rare, fatal inherited disorder of the nervous system that typically begins in childhood. The first symptom is usually progressive vision loss in previously healthy children followed by personality changes, behavioral problems and slow learning. Seizures commonly appear within 2–4 years of vision loss. However, seizures and psychosis can appear at any time during the course of the disease. Progressive loss of motor functions (movement and speech) start with clumsiness, stumbling and Parkinson‐like symptoms; eventually, those affected become wheelchair‐bound, are bedridden, and die prematurely. [see “CLN3‐Clinical Data.” *The neuronal ceroid lipofuscinoses (Batten disease).* Ed. Mole, S.E., Williams R.E., Goebel H.H., Machado da Silva G., Cary, North Carolina, Oxford University Press, 2011. Pages 117–119. Print].

The most common name for the disease is Batten disease which was first used to describe the juvenile and presumably the CLN3 form (prior to the discovery of the gene). Today the term Batten is widely used in the US and UK to refer to all 13 forms of neuronal ceroid lipofuscinosis. The disease has also been called Batten‐Mayou disease, Batten‐Spielmeyer‐Vogt disease, CLN3‐related neuronal ceroid‐lipofuscinosis, juvenile Batten disease, Juvenile cerebroretinal degeneration, juvenile neuronal ceroid lipofuscinosis, Spielmeyer‐Vogt disease and the term adopted in 2012, CLN3 disease. Using the Basic Local Alignment Search (BLAST) tool to align regions of similarity between known biological sequences, investigators discovered that the CLN3 gene is highly conserved amongst *Homo sapiens, Canis Lupus familiaris, Mus musculus, Danio rerio, Drosophila melanogaster, Caenorhabditis elegans, Schizosaccharomyces cerevisiae, and Schizosaccharomyces pombe* (Altschul et al., 1997; Katz et al., 1999; Mitchell, Porter, Kuwabara, & Mole, 2001; Pearce & Sherman, 1997)*.* The National Center for Biotechnology Information (NCBI) Gene lists 179 orthologs discovered through comparison of known sequences.

### 2.3 Exon and Intron structure and alternative splice variants of *CLN3*

The *CLN3* gene on the p‐arm of chromosome 16 spanning bases 28,466,653 to 28,492,302 http://genome.ucsc.edu/cgi-bin/hgGene?db=hg19%26hgg_gene=CLN3 (Haeussler et al., 2019; Kent et al., 2002) https://www.ncbi.nlm.nih.gov/variation/view/ (version 1.5.6 last update July 17, 2017). The CLN3 protein coding sequence begins on exon two at base 544 and ends at the final exon of the mRNA.

The NCBI Reference Sequence (RefSeq) database reports six transcript variants for the CLN3 gene suggesting that alternative splicing affects exons and the 3′ and 5′ UTRs (Untranslated Region;(Kent et al., 2002; Pruitt et al., 2014, https://www.ncbi.nlm.nih.gov/variation/view/; version 1.5.6 last update July 17, 2017) of the genomic sequence. Most of the six isoforms contain between 13 and 16 exons; however, shorter isoforms are also reported in the Consensus CDS Protein Set. CLN3 isoform a consists of 438 amino acids (aa), and is encoded by the longest transcript variant 1 (NM_001042432.1) (Barnett, Pickle, & Elting, 1990) and 2 (NM_000086.2), 1915 bp and 1879 bp, respectively. Transcript variant 3 (NM_001286104.1) encodes for isofom b (NP_001273033.1) which lacks an alternate in‐frame exon resulting in a shorter protein of 414 aas. Variant 4 (NM_001286105.1) encodes for isoform c (NP_001273034.1) which begins with a downstream AUG (start codon) in the 5′UTR resulting in a different N‐terminus. Isoform c also lacks two exons, which gives rise to a truncated version of the protein consisting of only 338 aas. Isoform d (NP_001273038.1) encoded by transcript variant 5 (NM_001286109.1) contains variations in both 5′ and 3′ UTRs and lacks an in‐frame exon resulting in translation initiation at a downstream AUG and resultant protein of 360 aas. Similarly, isoform e (NP_001273039.1) encoded by variant 6 (NM_001286110.1) harbors alterations in the 5′ UTR and lacks an in‐frame exon leading to a delay in the initiation of translation and produces a protein of 384 aas.

RefSeq records are generated when there is experimental or published evidence in support of the full‐length product, whereas transcript alignments to the assembled genome indicate the possibility of a gene product. RefSeq records list 6 transcripts whereas Ensembl, which is not limited to proven transcripts, lists 64 potential transcripts for the *CLN3* gene, providing additional clues to *CLN3’s* spatiotemporal patterns of expression. However, neither tissue, development, nor disease‐specific studies have been completed to indicate which transcripts are expressed under various conditions (Zerbino et al., 2018).

#### 2.3.1 Impact of splice variants and Single Nucleotide Polymorphisms on the protein domain structure of CLN3

**Figure 2:**
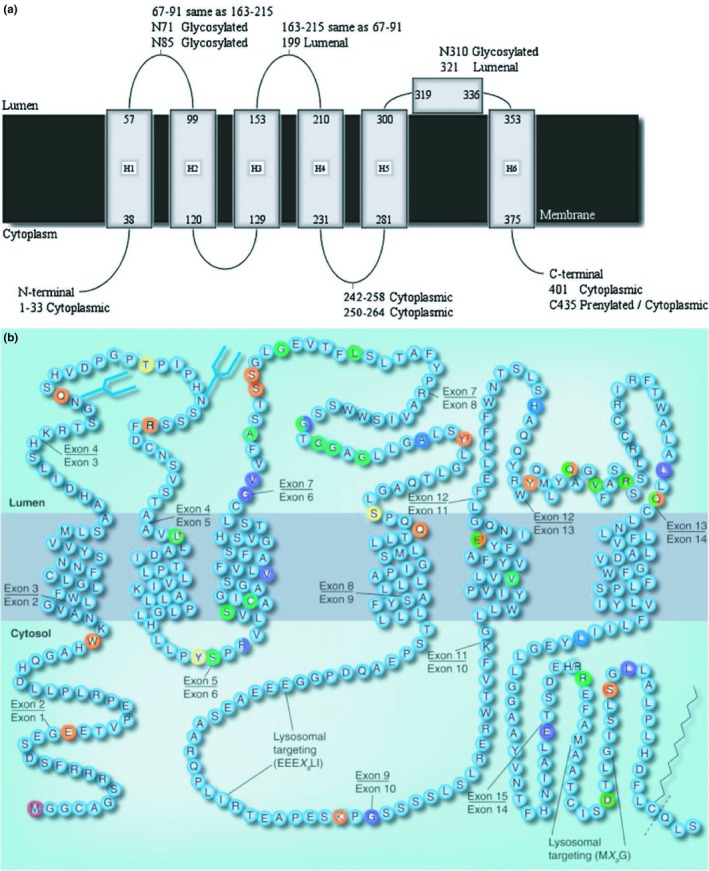
2a and 2b: 2a Schematic model for human CLN3 showing the six transmembrane helices, proposed amphipathic helix and experimentally determined loop locations. 2b The predicted topology of CLN3 is depicted. Sites for post‐translational modifications are shown as blue forked lines for N‐glycosylation, zigzags for prenylation, and dotted lines for potential cleavage sites following prenylation. Disease‐causing mutations are colored in red, orange, yellow, green, blue, and violet. If the mutation covers multiple residues, only the first residue is marked. (NB‐All disease‐causing mutations are not shown. For a completes list see Table 1)

Interestingly, mapping the mutations causative of Batten Disease on a CLN3 topological model reveals that most of these mutations face the luminal side of the intracellular compartments. Moreover, evolutionarily constrained analysis of the aa sequence revealed that luminal loop 2, is the most highly conserved domain across species (Gachet, Codlin, Hyams, & Mole, 2005; Muzaffar & Pearce, 2008). Of particular note, the most common mutation found in CLN3 disease patients, the “1kb” deletion in which exons 7 and 8 are excised, maps within this loop. Similar to the second loop, the predicted amphipathic helix on the luminal face between the fifth and sixth transmembrane helices contains several missense mutations (Figure 2) (Kousi, Lehesjoki, & Mole, 2012; Nugent, Mole, & Jones, 2008). The clustering of a majority of missense mutations in these two luminal regions strongly suggests that they are critical sites for CLN3 protein interaction and function (Cotman & Staropoli, 2012).

Mutations in *CLN3* are classically associated with CLN3 disease where retinal degeneration is followed by mental and physical deterioration and premature death. Recent studies show that CLN3 mutations may also result in a nonsyndromic retinal degeneration whose onset differs considerably from classical Batten disease. *CLN3* joins a growing number of genes such as TTC8, BBS2, and USH2A, whose mutations are commonly associated with syndromic diseases that include a subset of patients with isolated vision loss. Some mutations, as in the case of *CLN3,* can result in either phenotype (see Table 1) (Goyal, Jäger, Robinson, & Vanita, 2016; Rivolta, Sweklo, Berson, & Dryja, 2000; Shevach et al., 2015).

**Table 1:** Representation of reported disease‐causing mutations, their associated regions of the DNA (i.e. promoter, coding, noncoding), cDNA change, genomic DNA change, protein change, type of mutation, human phenotype (CLN3 disease or nonsyndromic retinitis pigmentosa). Adapted from https://www.ucl.ac.uk/ncl/CLN3mutationtable.htm

Amino Acid position	Mutation location	cDNA Change	Genomic DNA change	Protein change	Type of mutation	Phenotype	References
	Promoter	c.‐1101C>T	g.28504365G>A	p.(=)	Sequence variant		Kousi et al. (2012)
	Promoter	c.‐681_‐676delTGAAGC	g.28503756_28503761delGCTTCA	p.(?)	Sequence variant		Kousi et al. (2012)
1	Exon 1	c.1A>C	g.28503080T>G	p.?	Missense or aberrant start	CLN3 Disease	Kousi et al. (2012)
17	Exon 2	c.49G>T	g.28502879C>A	p.(Glu17*)	Nonsense		Kwon et al. (2005)
35	Exon 2	c.105G>A	g.28502823C>T	p.(Trp35*)	Nonsense	CLN3 Disease	Kousi et al. (2012)
	Exon 2	c.125+1G>C	g.28502794C>G	p.?	splice defect	Non‐syndromic retinal disease	Wang et al. (2014)
	Intron 2	c.125+5G>A	g.28502798C>T	p.?	splice defect	CLN3 Disease	Kousi et al. (2012)
	Intron 2	c.126‐1G>A	g.28500708C>T	p.?	splice defect	CLN3 Disease	Mole et al. (2001)
72	Exon 3	c.214C>T	g.28500619G>A	p.(Gln72*)	Nonsense	CLN3 Disease	Kousi et al. (2012)
	Intron 3	c.222+2T>G	g.28500609A>C	p.?	Splice site	CLN3 Disease	Kousi et al. (2012)
	Intron 3	c.222+5G>C	g.28500606C>G	p.?	Splice site	CLN3 Disease	Kousi et al. (2012)
80	Exon 4	c.233_234insG	g.28499972_28499973insC	p.(Thr80Asnfs*12)	Frameshift	CLN3 Disease	Kousi et al. (2012)
89	Exon 4	c.265C>T	g.28499941G>A	p.(Arg89*)	Nonsense	CLN3 Disease	Pérez‐Poyato et al. (2011)
101	Exon 5	c.302T>C	g.28499055A>G	p.(Leu101Pro)	Missense	CLN3 Disease, protracted	Munroe et al. (1997)
124	Exon 5	c.370dupT	g.28498987dup	p.(Tyr124Leufs*36)	Frameshift, introducing a premature stop codon	CLN3 Disease	Kousi et al. (2012)
	Exon 5 intron or Exon 6	c.375‐3C>G		p.(?)	splice	Non‐syndromic retinal disease	Ku et al. (2017)
127	Exon 6	c.378_379dupCC	g.28498856_28498857dup	p.(Arg127Profs*55)	1‐bp deletion and 2bp insertion	CLN3 Disease	Kousi et al. (2012); Munroe et al. (1997)
127	Exon 6	c.379delC	g.28498858delG	p.(Arg127Glyfs*54)	1‐bp deletion		Kousi et al. (2012)
131	Exon 6	c.391A>C	g.28498828T>G	p.(Ser131Arg)	Missense	Non‐syndromic retinal disease	Wang et al. (2014)
134	Exon 6	c.400T>C	g.28498837A>G	p.(Cys134Arg)	Missense	CLN3 Disease	Kousi et al. (2012)
142	Exon 6	c.424delG	g.28498813delC	p.(Val142Leufs*39)	1‐bp deletion	CLN3 Disease	Bensaoula et al. (2000), Kousi et al. (2012) and Munroe et al. (1997)
	maximum deletion of intron 5–15, Minimum deletion of exon 8–15;	c.432+?_1350‐?del	g.28488804_28498805del		6‐kb deletion	CLN3 Disease	Mitchison, O’Rawe, et al. (1995)
	Intron 6	c.461‐1G>C	g.28497972C>G	p.?	Splice defect	CLN3 Disease	Kousi et al. (2012)
	Intron 6	c.461‐3C>G		p.(?)	splice	Non‐syndromic retinal disease	Ku et al. (2017)
154	Intron 6 ‐ Intron 8	c.461‐280_677+382del966	g.28497286_28498251del	p.[Gly154Alafs*29, Val155_Gly264del]	(966 b deletion)		Batten Disease Consortium (1995), Munroe et al., (1997), Wisniewski, Zhong, et al. (1998), Zhong et al. (1998), Lauronen et al. (1999), Eksandh et al., 2000, Bensaoula et al., 2000, Teixeira et al. (2003), de los Reyes et al. (2004), Leman, Pearce, and Rothberg (2005), Kwon et al. (2005), Moore et al. (2008), Pérez‐Poyato et al. (2011), Kousi et al. (2012) and Ku et al. (2017)
154	Intron 6 ‐ Intron 8	c.461‐280_677+382del966	g.28497286_28498251del	p.[Gly154Alafs*29, Val155_Gly264del]	(966 b deletion)	Non‐syndromic retinal disease	Ku et al. (2017)
155	Intron 6	c.461‐13G>C	g.28497984C>G	p.[=, Val155Profs*2]	Aberrant splicing that removes exon 7	CLN3 Disease	Munroe et al. (1997)
158	Exon 7	c.472G>C	g.28497960C>G	p.(Ala158Pro)	Missense	CLN3 Disease	Kousi et al. (2012)
161	Exon 7	c.482C>G	g.28497950G>C	p.(Ser161*)	Nonsense		Munroe et al. (1997)
162	Exon 7			p.(Ser162*)	Nonsense		Munroe et al. (1997)
165	Exon 7	c.494G>A		p.(Gly165Glu)	Missense		Cortese et al. (2014)
170	Exon 7	c.509T>C	g.28497923A>G	p.(Leu170Pro)	Missense	CLN3 Disease, protracted	Munroe et al. (1997)
	Intron 7	c.533+1G>C	g.28497898C>G	p.?	Splice defect/frameshift	CLN3 Disease, protracted	Batten Disease Consortium (1995), Lauronen et al. (1999) and Munroe et al. (1997)
	Intron 7	c.533+1G>A	g.28497898C>T	p.?	Splice site	CLN3 Disease	Kousi et al. (2012)
187	Exon 8	c.558_559delAG	g.28497786_28497787delCT	p.(Gly187Aspfs*48)	2‐bp deletion and Missense	CLN3 Disease	Munroe et al. (1997)
187	Exon 8	c.560G>C	g.28497785C>G	p.(Gly187Ala)	Missense	CLN3 Disease	Mole et al. (2001) and Kousi et al. (2012)
189	Exon 8	c.565G>C	g.28497780C>G	p.(Gly189Arg)	Missense	CLN3 Disease and Non‐syndromic retinal dystrophy	Kousi et al. (2012) and Wang et al. (2014)
189	Exon 8			p.(Gly189Arg)	Missense	CLN3 Disease and Non‐syndromic retinal dystrophy	Kousi et al. (2012); Wang et al. (2014)
192	Exon 8	c.575G>A	g.28497770C>T	p.(Gly192Glu)	Missense	CLN3 Disease	Pérez‐Poyato et al. (2011)
	Exon 8	c.582G>T	g.28497763C>A	p.(=)	Sequence variant	CLN3 Disease, protracted	Sarpong et al. (2009)
196	Exon 8	c.586dupG or c.586‐587insG	g.28497759dup	p.(Ala196Glyfs*40)	1‐bp insertion	CLN3 Disease	Munroe et al. (1997)
199	Exon 8	c.597C>A	g.28497748G>T	p.(Tyr199*)	Nonsense	CLN3 Disease, protracted	Sarpong et al. (2009), Kousi et al. (2012) and Mole et al. (2001)
208	Exon 8	c.622dupT	g.28497723dup	p.(ser208Phefs*28)	Frameshift, introducing a premature stop codon		Kousi et al. (2012) and Pérez‐Poyato et al. (2011)
211	Exon 8	c.631C>T	g.28497714G>A	p.(Gln211*)	Nonsense		Munroe et al. (1997)
	Intron 8	c.678‐?_1317+?del	g.28488837‐?_28495439+?del	p.?	partly characterised deletion	CLN3 Disease	Kousi et al. (2012)
262	Exon 9	c.784A>T		p.(Lys262*)	Nonsense	CLN3 Disease	Coppieters et al. (2014)
	Intron 9	c.790+3A>C	g.28495324T>G	p.?	Sequence Variant	CLN3 Disease	Kousi et al. (2012)
264	Intron 9 ‐ Intron 13	c. 791_1056del	g.28491981_28494795del2815	p.(Gly264Valfs*29)	2.8‐kb deletion	CLN3 Disease, protracted	Batten Disease Consortium (1995), Munroe et al. (1997), Lauronen et al. (1999), Kousi et al. (2012) and Mole et al. (2001)
155	Exon 10	c.831G>A	g.28493953C>T	p.[Val155_Gly264del, Gly280_Leu302del]	Sequence variant	CLN3 Disease	Zhong et al. (1998)
	Exon 10 intron or Exon 11	c.837+5G>A		p.(?)	splice	CLN3 Disease and Non‐syndromic retinal dystrophy	Ku et al. (2017)
285	Exon 11		g.28493851T>C	p.(Ile285Val)	missense	CLN3 Disease and Non‐syndromic retinal dystrophy	Carss et al. (2017) and Ku et al. (2017)
290	Exon 11	c.868G>T		p.(Val290Leu)	Missense	Non‐syndromic retinal disease	Wang et al. (2014)
295	Exon 11	c.883G>A	g.28493821C>T	p.Glu295Lys	Missense and Nonsense	CLN3 Disease, protracted	Lauronen et al. (1999), Munroe et al. (1997), Wang et al. (2014) and Zhong et al. (1998)
295	Exon 11	c.883G>T	g.28493821C>A	p.(Glu295*)	nonsense	CLN3 Disease and Non‐syndromic retinal dystrophy	Kousi et al. (2012), Ku et al. (2017) and Mole et al. (2001)
	Intron 11	c.906+5G>A	g.28493793C>T	splice defect	Splice site	CLN3 Disease	Kousi et al. (2012)
306	Exon 12	c.917T>A		p.(Leu306His)	missense	Non‐syndromic retinal disease	Ku et al. (2017)
	Exon 12	c.944‐945insA				CLN3 Disease	Munroe et al. (1997)
313	Exon 12	c.954_962+18del27	g.28493630_28493656del27	p.Leu313_Trp321del/ splice defect	Splice defect	CLN3 Disease	Kousi et al. (2012)
315	Exon 12	c.944dupA	g.28493666dup	p.(His315Glnfs*67)	1‐bp insertion	CLN3 Disease	Licchetta et al. (2015)
	Exon 12	c.10633‐10660del			Frameshift after Q318, possible aberrant splicing		Mole et al. (2001)
	Exon12‐Intron12	c.963‐1G>T	g.28493520C>A	p.?	Splice defect	CLN3 Disease	Kousi et al. (2012)
322		c.966C>G		p.(Tyr322*)	Nonsense	Non‐syndromic retinal disease	Wang et al. (2014)
330	Exon 13	c.988G>A	g.28493494C>T	p.(Val330Phe)	Missense	Non‐syndromic retinal disease	Carss et al. (2017) and Ku et al. (2017)
334	Exon 13	c.1000C>T	g.28493482G>A	p.Arg334Cys	Missense	CLN3 Disease	Kousi et al. (2012) and Munroe et al. (1997)
334	Exon 13	c.1001G>A	g.28493481C>T	p.Arg334His	Missense	CLN3 Disease, protracted	Batten Disease Consortium (1995), Kousi et al. (2012), Munroe et al. (1997) and Pérez‐Poyato et al. (2011)
349	Exon 13	c.1045_1050del		p.(Ala349_Leu350del)	6‐bp deletion	CLN3 Disease	Licchetta et al. (2015)
350	Exon 13	c.1048delC	g.28493434delG	p.Leu350CysfsX27	1‐bp deletion	CLN3 Disease	Kousi et al. (2012)
352	Exon 13	c.1054C>T	g.28493428G>A	p.Gln352X	Nonsense	CLN3 Disease	Kousi et al. (2012) and Munroe et al. (1997)
	Intron 13	c.1056+3A>C	g.28493423T>G	p.?	Splice site	CLN3 Disease	Kousi et al. (2012)
379	Exon 14	c.1135_1138delCTGT		p.(Leu379Metfs*11)	4‐bp insertion	CLN3 Disease	Drack, Miller, and Pearce (2013)
399	Exon 14	c.1195G>T	g.28489060C>A	p.(Glu399*)	27‐bp deletion	CLN3 Disease	Kousi et al. (2012)
400	Intron 14	c.1198‐1G>T	g.28488957C>A	p.Thr400*	Splice site	CLN3 Disease	Munroe et al. (1997); Mole et al. (2001)
404	Exon 15	c.1211A>G	g.28488943T>C	p.(His404Arg)	Polymorphism	CLN3 Disease	Munroe et al. (1997) and Mole et al. (2001)
405	Exon 15	c.1213C>T	g.28488945G>A	p.(Arg405Trp)	Missense	Non‐syndromic retinal disease	Carss et al. (2017), Ku et al. (2017), Wang et al. (2014) and Weisschuh et al. (2016)
416	Exon 15	c.1247A>G	g.28488907T>C	p.(Asp416Gly)	Missense	CLN3 Disease	Kousi et al. (2012)
423	Exon 15	c.1268C>A	g.28488886G>T	p.(Ser423*)	Nonsense	CLN3 Disease	Kousi et al. (2012)
425	Exon 15	c.1272delG	g.28488882delC	p.(Leu425Serfs*87)	1‐bp deletion	CLN3 Disease	Munroe et al. (1997)
			g.28497285‐28498251del	NA	large deletion	Non‐syndromic retinal disease	Carss et al. (2017)

### 2.4 *CLN3* gene regulation

The *CLN3* gene was sequenced in 1997 following the discovery of the protein. Sequencing began 1.1 kb upstream of the transcription start site and proceeded to 0.3 kb downstream of the polyadenylation site. Sequencing led to the conclusion that *CLN3* is organized into at least 15 exons spanning 15 kb ranging from 47 to 356 bps in length (Mitchison et al., 1997). *CLN3* also has 14 introns that vary from 80 to 4,227 bps in length. There are a total of 12 *Alu* repeats in the forward orientation and 9 in reverse orientation present within the introns and 5′‐ and 3′‐untranslated regions. The 5′ region of the *CLN3* gene contains several potential transcription regulatory elements. Although there are two (TA)*_n_* repetitive motifs at nt 135 and 335 in the sequence, there is no consensus TATA‐1 box evident, suggesting that *CLN3* is constitutively expressed (Mitchison et al., 1997). In addition, using the transcription factor DNA binding site database, putative *cis*‐acting regulatory elements were found in the 5′ flanking sequence, including potential transcription factor binding sites for AP‐1, AP‐2, and Sp1, two motifs for the erythroid‐specific transcription factor GATA‐1 and three potential CCAAT boxes (Mitchison et al., 1997).

#### 2.4.1 CLN3 promotor and transcription factor binding analysis

Several regulatory elements have been found in the 5′ region of the *CLN3* gene; however, the endogenous promoter of *CLN3* has not been definitively characterized yet. To this end, Eliason and colleagues created transgenic CLN3 mice by knocking‐in a DNA sequence encoding for nuclear‐targeted bacterial β‐gal. This was achieved via homologous recombination of a targeting construct into embryonic stem cells, such that β‐gal transcription was controlled by a native sequence 5′ to the *CLN3* coding region. This resulted in the replacement of most of exon 1 and all of exons 2–8, creating an effective null mutation (Ding, Tecedor, Stein, & Davidson, 2011; Eliason et al., 2007). In these studies, the authors demonstrated that *CLN3* is ubiquitously expressed and that a regulatory and functional region at the 5′ of the gene is promoting its expression. Other studies have described a putative promoter region and identified predicted transcription factor binding sites (see Table 2 for complete list). Moreover, a multitude of transcription factors have been reported to regulate *CLN3* expression pattern by direct interaction (physical binding) with the “promoter” region or by indirect interaction through protein partners. Indeed, TFEB has been shown to bind to CLEAR elements on the proximal promoter of CLN3, which increases CLN3 transcription (Palmieri et al., 2011; Sardiello et al., 2009). TFEB binding sites were first mapped to −24 (AGCACGTGAT) and +6 (GTCACGTGAT) on the promoter of CLN3 (Sardiello et al., 2009) and physical binding of TFEB was then further experimentally verified by ChIP‐seq (Palmieri et al., 2011). Table 2 reports nonredundant transcription factor interactor of *CLN3* that regulate its expression profile. Additional information on each interaction, that is, the type of experimental support and key functional statement, is provided below.

**Table 2:** Transcriptional regulators of CLN3

Putative Transcriptional Regulator	Protein details	Putative effect	Methods used to identify putative transcriptional regulator	References
AP‐2A	Putative CLN3 promoter has an AP‐2A binding site.	Putative CLN3 promoter has an AP‐2A binding site.	The authors developed a computational genomics strategy termed ChIPModules which begins with experimentally‐determined binding sites and integrates those with positional weight matrices constructed from transcription factor binding sites, comparative genomics, and statistical learning methods with the purpose of identifying transcriptional regulatory modules. They identify that the putative CLN3 promoter contains an AP‐2alpha binding site	Jin, Rabinovich, Squazzo, Green, and Farnham (2006)
E2F1	E2F1_HUMAN	Putative CLN3 promoter has a putative E2F1 binding site.	Researchers studied the binding of E2F1 to promoters. ChIP analyses of 24,000 promoters confirmed that more than 20% of promoters are bound by E2F1. Including the CLN3 promoter	Bieda, Xiaoqin, Singer, Green, and Farnham (2006)
ELAV1 (huR)E2F1	E2F1_HUMAN	Putative CLN3 promoter has a putative E2F1 binding site.	HuR regulates the stability and translation of numerous mRNAs encoding for stress response and proliferative proteins. HuR was found to bind CLN3 mRNA. The interaction value was higher than the mock controls; albeit very weak. Researchers studied the binding of E2F1 to promoters. ChIP analyses of 24,000 promoters confirmed that more than 20% of promoters are bound by E2F1. Including the CLN3 promoter	Abdelmohsen et al. (2009); Bieda et al. (2006)
ETS1ELAV1 (huR)	ETS1 Human	ETS1 binds to the putative CLN3 promoter	The CLN3 promoter contains putative ETS1 binding motifs. HuR regulates the stability and translation of numerous mRNAs encoding for stress responses and proliferative proteins. HuR was found to bind CLN3 mRNA. The interaction value was higher than the mock controls; albeit very weak.	Hollenhorst et al. (2009); Abdelmohsen et al. (2009)
ETS1ETS1	ETS1 MouseETS1 Human	ETS1 binds to the putative CLN3 promoter	CLN3 is downregulated in ETS1‐/‐ mNK cells. The CLN3 promoter contains putative ETS1‐binding motifs.	Ramirez et al. (2012); Hollenhorst et al. (2009)
HNF4‐alphaETS1	HNF4A_HUMANETS1 Mouse	HNF4‐alpha binds to gene CLN3 promoterETS1	In the referenced paper, CLN3 appears in a list of potential HNF4alpha target genes in differentiated Caco2 colorectal adenocarcinoma cells. CLN3 is downregulated in ETS1‐/‐ mNK cells	Bolotin et al. (2010), Boyd, Bressendorff, Møller, Olsen, and Troelsen (2009) and Ramirez et al. (2012)
HSF1HNF4‐alpha	HSF1_HUMANHNF4A_HUMAN	CLN3 harbors an HSF 1 site I at a proximal Alu in an antisense orientationHNF4‐alpha binds to gene CLN3 promoter	CLN3 is downregulated upon heat shock in microarray experiments. In the referenced paper, CLN3 appears in a list of potential HNF4alpha target genes in differentiated Caco2 colorectal adenocarcinoma cells.	Bolotin et al. (2010), Boyd et al. (2009) and Pandey, Mandal, Jha, and Mukerji (2011)
TFEB	CLEAR	CLN3 harbors a two CLEAR binding site on its proximal promoter (at −24 nad +6).	TFEB has been shown to bind this (CLEAR) element in the proximal promoter of CLN3 and increase CLN3 transcription.	Palmieri et al. (2011) and Sardiello et al. (2009)

Additionally, transcription factor binding sites were predicted for the *CLN3* promoter using the sequence‐based profiles of known sites. Position‐specific scoring matrices were applied for 202 human transcription factors or factor dimers obtained from the JASPAR database (Portales‐Casamar et al., 2010). The promoter region of *CLN3* was obtained from the UCSC Genome Browser using the RefSeq gene boundaries (Pruitt et al., 2014). A 1‐kb region upstream and 500 bases downstream of the transcription start site (TSS) were used for this analysis. The motif search was performed using the MAST tool from the MEME suite (Bailey et al., 2009).

The complete list of predicted transcription factor binding sites is shown in Table 3. Interestingly, the transcription factors SP2, ESRRA, Klf4, and USF2 each have three or more predicted binding sites in the vicinity of the transcription start site of *CLN3*.

**Table 3:** Predicted transcription factor binding sites on the CLN3 gene

Position Relative to TSS	Transcription factor	*p*‐value	Length
−329/−123	E2F3	6.9E‐05/5.6E‐07	14
−323	E2F4	8.50E‐05	10
−734	EGR2	5.90E‐05	14
−549	ELF1	3.40E‐06	12
−1,396	ESR1	3.50E‐05	19
−1,344/−672/−214	ESRRA	7.4E‐05/‐9.10E‐05/3.05E‐05	10
−919	FOXA1	1.40E‐06	14
−915	Foxa2	8.20E‐08	11
−131	FOXC1	5.00E‐05	7
−1,276	Foxd3	3.20E‐05	11
−777	FOXI1	9.80E‐05	11
−1,469/−1,271/−807	FOXP1	3.7E‐05/2.2E‐05/3.00E‐05	14
−545	GABPA	5.50E‐06	10
−1,364	Gata4	4.40E‐05	10
−603/−407/−388	Klf4	1.4E‐05/3.60E‐05/5.00E‐07	9
−1,222	Meis1	9.90E‐05	14
−83	NHLH1	7.50E‐05	11
−1,349/−677	NR2F1	5.4E‐05/2.00E‐05	13
−172	NRF1	9.60E‐05	10
−495	Pax2	9.70E‐05	7
−1,468	Pax4	2.80E‐05	29
−1,493/−1,102	PAX5	2.2E‐06/4.40E‐05	18
−1,317	PBX1	4.60E‐05	11
−230	PLAG1	1.60E‐06	13
−1,285	POU2F2	3.90E‐05	12
−1,253	PRDM1	8.20E‐06	14
−1,136	Rfx1	7.60E‐05	13
−1,167	RFX5	6.30E‐05	14
−603/−412/−393/−123	SP2	1.30E‐05/1.90E‐05/6.80E‐05/4.60E‐06	14
−715/−305	Tcfcp2l1	8.90E‐05/5.70E‐05	13
−1,409/−434	TFAP2C	5.70E‐05/1.60E‐05	14
−711	THAP1	4.60E‐05	8
−308	TP63	2.70E‐05	19
−786/−494/−267	USF2	8.00E‐05/7.60E‐06/7.20E‐05	10
−242	ZBTB33	8.80E‐05	14
−83	ZEB1	4.80E‐05	8
−433	Zfx	2.60E‐05	13

Note

Transcription Factors from MetaCore from Clarivate Analytics (Ekins, Nikolsky, Bugrim, Kirillov, & Nikolskaya, 2007).

In addition to regulation by transcription factors, studies have demonstrated that various molecules can indirectly regulate the expression of CLN3. These are listed in Table 4.

**Table 4:** Alternative transcriptional regulation

Alternative Transcription Regulator	Protein details		Methods used to Identify Putative Transcriptional Regulator	PubMed ID
*LactoferrinHSF1*	*TRFL_HUMAN DeltaLfHSF1_HUMAN*	*CLN3 harbors an HSF 1 site I at a proximal Alu in an antisense orientation*	*One form of LF is secreted in body fluids (sLF) whereas an alternative form deltal LF, regulated by a different promoter, is present in normal tissues. Delta LF is downregulated in breast cancer. The CLN3 promoter is downregulated in delta LF expressing HEK cell upon heat shock in microarray experiments.*	Kim, Kang, and Kim (2013) and Pandey et al. (2011)
*MITFLactoferrin*	*MITF_HUMANTRFL_HUMAN DeltaLf*	*MITF interacts with the putative CLN3 promoter*	*Putative CLN3 promoters have been pulled down by gene‐wide chromatin immunoprecipitation of MITF. One form of LF is secreted in body fluids (sLF) whereas an alternative form deltal LF, regulated by a different promoter, is present in normal tissues. Delta LF is downregulated in breast cancer. CLN3 is downregulated in delta LF expressing cell's.promoter.*	Bertolotto et al. (2011) and Kim et al. (2013)
*N‐MyristoylationN‐Myc*	*N‐myristoyltransferase_(HUMAN)MYCN_MOUSE*	*N‐Myc regulates transcription of CLN3*	*Using SILAC, researchers found that CLN3 is N‐myristoylated. The authors studied changes in gene expression in embryonic stem cells after the induction of various transcription factors. CLN3 was included in the analyses.*	Chen et al. (2008) and Nishiyama et al. (2009)

### 2.5 Description of the CLN3 protein

The *CLN3* gene encodes a highly hydrophobic protein of 438‐amino acids, the three‐dimensional structure of which has not yet been proven through X‐ray crystallography or Nuclear Magnetic Resonance‐spectroscopy. The secondary structure of CLN3 is mainly comprised of transmembrane and low complexity cytosolic or luminal spans (Berman et al., 2000). Computer prediction models suggest CLN3 spans the membrane anywhere from five to ten times (SPEP + Ensemble 1.0, MEMSAT3 + Swissprot, MEMSAT3, TMHMM 2.0, PHOBIUS Constrained, PROFPHD, HMMTOP). However, some experimental studies support a structure where the N‐terminus faces the intraluminal space of organelles with the C‐terminus facing the cytoplasm, the most widely cited peer‐reviewed articles favor a 6‐membrane spanning domain (MSD) model with both the N‐ and C‐termini facing the cytosol. See Figure 2 table below for more detailed information (Ezaki et al., 2003; Mao, Foster, Xia, & Davidson, 2003; Mao, Xia, & Davidson, 2003; Ratajczak, Petcherski, Ramos‐Moreno, & Ruonala, 2014).

**Table 5:** Locations of experimentally determined regions/residues

Region/residue	Location	References
N‐terminal	Cytoplasmic	Ezaki et al. (2003) and Ratajczak et al. (2014)
1–33	Cytoplasmic	
2–18	Lumenal	Mao, Foster, et al. (2003) and Mao, Xia, et al. (2003)
N71	Lumenal	
N85	Lumenal	Storch et al. (2007)
*97–121	MSD	[SPEP + Ensemble 1.0, MEMSAT3, + Swissprot, MEMSAT3 + CLN3, TMHMM 2.0, PHOBIUS Constrained, PROFPHD, HMMTOP]
199	Lumenal	Mao, Foster, et al. (2003)
210–231	MSD	[SPEP + Ensemble 1.0, MEMSAT3, + Swissprot, MEMSAT3 + CLN3, TMHMM 2.0, PHOBIUS Constrained, PROFPHD, HMMTOP]
250–264	Cytoplasmic	Mao, Foster, et al. (2003) and Mao, Xia, et al. (2003)
242–258	Cytoplasmic	Kyttälä et al. (2004)
*276–303	MSD	[SPEP + Ensemble 1.0, MEMSAT3, + Swissprot, MEMSAT3 + CLN3, TMHMM 2.0, PHOBIUS Constrained, PROFPHD, HMMTOP]
N310	Lumenal	Mao, Foster, et al. (2003) and Storch et al. (2007)
321	Lumenal	Mao, Foster, et al. (2003)
S401	Cytoplasmic	Kyttälä et al., (2004) and Ratajczak et al. (2014)
406–433	Cytopasmic	Nugent et al. (2008)
Cys435	Cytoplasmic	Storch et al. (2007)
C‐terminal	Cytoplasmic	Mao, Xia, et al. (2003) and Ratajczak et al. (2014)

* Computer Modeling was included for MSDs for which all prediction models support the same conclusion.

### 2.6 CLN3 protein details

#### 2.6.1 Consensus sequence elements similar to other proteins

The CLN3 protein sequence does not display significant similarities to any protein of known function (Muzaffar & Pearce, 2008). However, some consensus sequence elements within the CLN3 protein allude to its localization, regulation, and function. Early predictions of CLN3 using Position‐Specific Iterative Basic Local Alignment Search Tool (PSI‐BLAST) revealed a distant but significant sequence similarity between CLN3 and members of the SLC29 family of equilibrative nucleoside transporters, of which four members are recognized in mammals (Altschul et al., 1997; Baldwin et al., 2004). More recent algorithms, such as the Structural Classification of Proteins (Andreeva et al., 2008) and Protein families (Pfam) suggest that most of the CLN3 protein (aa 11–433) has a domain structure consistent with members of the major facilitator superfamily (MFS; SCOP superfamily 103473; Pfam clan CL0015). The MFS superfamily is one of the two largest families of membrane transporters and includes small‐solute uniporters, symporters and antiporters (Marger & Saier, 1993). Structural similarities between CLN3 and MFS family member MFSD8, may provide important clues as to its function. Indeed, mutations in MFSD8, which encodes a lysosomal protein with 12‐predicted transmembrane domains and unknown function, result in histological and phenotypical similarities to another form of Batten disease, CLN7, (Siintola et al., 2007). Moreover, sequence alignment and Markov modeling predicted the N‐terminus of CLN3 to be weakly homologous to fatty acid desaturases. Using nervous system and pancreatic tissue samples from a murine homozygous‐knockout model of CLN3, investigators demonstrated that Δ9 desaturase activity was greatly reduced, while heterozygous carriers displayed intermediate desaturase levels (40%) compared to wild‐type animals. Therefore, the loss of CLN3 appears to result in decreased desaturase activity on palmitoyl (C16) moieties of protein substrates (Narayan, Rakheja, Tan, Pastor, & Bennett, 2006; Narayan, Tan, & Bennett, 2008).

Sequence analysis also indicated a multitude of putative trafficking and sorting signals, suggesting CLN3 may populate a variety of organelles. A mitochondrial targeting signal was identified at residue 11 with a cleavage site at residue 19 (Janes et al., 1996). Furthermore, targeting studies demonstrated the existence of two lysosomal sorting signals; (a) a conventional dileucine motif preceded by an acidic patch located in a putative cytosolic loop of the favored 6‐transmembrane structure (Kyttälä et al., 2004) at 242EEE(X)_8_LI254 and (b) an unconventional motif in the long C‐terminal cytosolic tail consisting of methionine and glycine separated by nine amino acids [*M*(X)9G] (Kyttälä et al., 2005, 2004; Järvelä et al., 1998; Kida et al., 1999; Storch, Pohl, & Braulke, 2004). Interestingly, green fluorescent protein (GFP)‐tagged CLN3 with a double mutation in the dileucine motif (Leu425Leu426), a putative lysosomal targeting motif, to glycine (Gly425Gly426) still co‐localized with lysosomal associated membrane protein‐1 (LAMP1) in chinese hamster ovary (CHO) cells, suggesting that the dileucine motif is not required for the targeting of CLN3 to the lysosome. Since the dileucine motif is conserved among species, these results suggest that CLN3 contains additional lysosomal targeting sequences or different lysosomal targeting signals altogether (Kida et al., 1999). In contrast truncations of CLN3: GFP‐CLN3(1–322), GFP‐CLN3(138–438), and CLN3(1–138)‐GFP do not localize to lysosomes (Kida et al., 1999) indicating that the missing regions either contain imperative lysosome targeting signals or their absence alters the 3D structure of the protein.

Initial studies suggested that yeast CLN3 homolog Btn1 in *S. cerevisiae* and *S. pombe* localizes to yeast vacuoles (Croopnick, Choi, & Mueller, 1998; Gachet et al., 2005; Pearce, Ferea, Nosel, Das, & Sherman, 1999; Wolfe, Padilla‐Lopez, Vitiello, & Pearce, 2011). However, more recent studies suggest that experimental tags may have mislocalized the protein. Indeed, when not tagged at its C‐terminus, Btn1 localizes to the Golgi apparatus (Codlin & Mole, 2009; Dobzinski, Chuartzman, Kama, Schuldiner, & Gerst, 2015; Kama, Kanneganti, Ungermann, & Gerst, 2011; Vitiello, Benedict, Padilla‐Lopez, & Pearce, 2010).

#### 2.6.2 Post‐translational modifications of the CLN3 protein

CLN3 contains several putative post‐translational modification (PTM) motifs which contribute to the targeting and anchoring of CLN3 to distinct biological membranes (Casey, 1995). These motifs include four putative N‐glycosylation sites, two putative O‐glycosylation sites, and consensus sequences for phosphorylation, myristoylation, and farnesylation (Ellgaard & Helenius, 2003; Golabek et al., 1999; Haskell, Carr, Pearce, Bennett, & Davidson, 2000; Järvelä et al., 1998; Kaczmarski et al., 1999; Kida et al., 1999; Mao, Xia, et al., 2003; Michalewski et al., 1998, 1999; Nugent et al., 2008; Pullarkat & Morris, 1997; Sigrist et al., 2013; Storch, Pohl, Quitsch, Falley, & Braulke, 2007; Taschner, de Vos, & Breuning, 1997b).

##### Glycosylation

Alignment and comparison of the CLN3 amino acid sequences across species (human, canine, murine, and yeast CLN3; Genbank Accession number U32680, L76281.1, U68064, AF058447.1) revealed a number of highly conserved N‐X‐S/T motifs, indicating conservation of putative glycosylation sites. In vitro translation of CLN3 produced a singlet at 43 kilodaltons (kDa) in the absence of microsomal membranes and a doublet at 43 and 45 kDa in the presence of microsomal membranes (using rabbit antibody 385/CLN3 raised against residues 242–258 [EEEAESAARQPLIRTEA], which map to the long cytosolic loop according to the most cited prediction model). Similarly, intracellular synthesis and maturation of CLN3 in COS‐1 and HeLa cells also identified the 43 kDa nonglycosylated and a 45 kDa glycosylated forms of CLN3. In detail, pulse‐chase of transfected COS‐1 cells followed by immunoprecipitation showed a single band of 43 kDa 1 hr following the pulse, while further chase up to 6 hr revealed a characteristic doublet of 43 and 45 kDa. Human N‐glycosylated CLN3 protein is sensitive to endoglycosidase H suggesting a high‐mannose type glycosylation (Järvelä et al., 1998). In contrast, murine CLN3 contains complex‐type N‐linked sugars that differ from the human CLN3 (Ezaki et al., 2003). Moreover, mass spectrometric analyses revealed that CLN3 exhibits tissue‐dependent glycosylation patterns (Ezaki et al., 2003). Thus, the apparent molecular weight of glycosylated CLN3 protein may vary depending on cell type and species (Golabek et al., 1999).

Expression of GFP‐CLN3 fusion protein resulted in a 66 and a 100 kDa bands in neuroblastoma and CHO cells, whereas in COS and HeLa cells only the 66 kDa band is detectable. Expression of GFP alone resulted in a 27kDa band, indicating that CLN3 alone would result in ~39 and ~73 kDa bands, respectively. Pulse‐chase experiments revealed that the 66 kDa form appears first, followed by the 100 kDa band. Both the 66 and 100 kDa forms are digested by complex oligosaccharide amidase Peptide ‐*N*‐Glycosidase F down to 64 kDa. Whereas glycosidase Endoglycosidase H only digests the 66 kDa form. Thus, the 100 kDa form is a complex oligosaccharide in some cell types (Golabek et al., 1999).

N‐linked glycosylation of integral membrane proteins in the ER and in the early secretory pathways, has been shown to be important for protein folding, oligomerization, quality control, sorting and function (Ellgaard & Helenius, 2003). Human CLN3 possesses four potential N‐glycosylation sites (N49, N71, N85, and N310). Glycosylation of N49 is physically unlikely because this residue is located in the first membrane domain. Mutational analyses demonstrated that N71 and N85, located in the first luminal domain, are N‐linked glycosylated (Storch et al., 2007). It remains unclear whether N310, in the third luminal domain, is N‐linked glycosylated (Mao, Foster, et al., 2003; Storch et al., 2007). N‐glycosylation is not required for the proper trafficking of CLN3, as neither treatment with the N‐glycosylation inhibitor tunicamycin, nor single or double substitution of N71 and N85 affected the stability or the trafficking of CLN3 to lysosomes (Golabek et al., 1999; Kida et al., 1999; Storch et al., 2007).

CLN3 also possesses two putative O‐glycosylation sites at T80 and T256 (Consortium 1995). However, O‐glycosylation sites are poorly defined, not necessarily utilized, and T256 is predicted to be cytoplasmic, which is not compatible with glycosylation.

##### Phosphorylation

Sequence analyses using the ScanPROSITE tool (Sigrist et al., 2013) suggest that CLN3 contains nine putative phosphorylation sites: six on cytoplasmic loops (Ser12, Ser14, Thr19, Thr232, Ser270, Thr400) and three on luminal loops (Ser69, Ser74, Ser86) (Nugent et al., 2008). Previous computer‐based predictions identified 10 serine and 3 threonine residues that may undergo phosphorylation (Michalewski et al., 1998). GFP‐CLN3 expressed in CHO cells incorporates 32P in both the 66 and 100 kDa forms, when incubated with cAMP‐dependent protein kinase (PKA), cGMP‐dependent protein kinase (PKG) or casein kinase II. The reaction was reversed by alkaline phosphatase, indicating that GFP‐CLN3 is indeed phosphorylated (Michalewski et al., 1998, 1999) by PKA, PKG, and casein kinase II and can be enhanced by inhibition of protein phosphatase 1 or protein phosphatase 2A. However, as these studies relied solely on in vitro assays using kinase activators or phosphatase inhibitors, future studies based on protein knockdown in cellular systems will be useful to better assess the specificity and the biological role of each of these proteins in the regulation of CLN3. However, phosphorylation is important for multiple physiological functions such as membrane targeting, protein–protein interactions and the formation of functional complexes, the phosphorylation states and significance of CLN3 phosphorylation remain elusive. The generation of CLN3 phosphomutants would have merit for fully elucidating the biological relevance of these PTMs.

##### Myristoylation

A putative N‐myristoylation site exists at 2**GGCAGS**7 in human (Genbank Accession number U32680), canine (Genbank accession number L76281.1) and murine (Genbank Accession number U68064) CLN3. The significance of this lipid modification to CLN3 has not been explored experimentally. However, covalent attachment of myristoyl group by an amide bond to an alpha‐amino group of a N‐terminal glycine has been implicated in protein‐protein and protein‐lipid interactions, membrane targeting, and numerous signal transduction steps. Conservation of N‐myristoylation motifs in human, dog, and mouse as well as isoprenylation motifs in human, dog, mouse and yeast suggest that CLN3 could be a membrane‐attached protein despite the lack of a signaling peptide (Taschner, de Vos, & Breuning, 1997a).

##### Prenylation/Farnesylation

Prenylation refers to the addition of hydrophobic molecules to a substrate, and involves the transfer of either farnesyl or geranyl‐geranyl moiety to C‐terminal cysteine(s) of the target protein. It is believed that prenyl group modifications facilitate attachment to cell membranes, similar to lipid anchors. Farnesylation is a type of prenylation, where an isoprenyl group is added to a cysteine residue. These modifications are important for protein–protein and protein–membrane interactions. Sequence analyses of CLN3 predict a CAAX motif 435CQLS438 at the C‐terminus that can be prenylated (Taschner et al., 1997a). Coupled translation/prenylation reactions of CLN3 and tetra‐peptides in vitro demonstrate that the CQLS sequence acts as a good acceptor for a farnesylation group (Kaczmarski et al., 1999; Pullarkat & Morris, 1997). Furthermore, glutathione S transferase (GST)‐fusion CLN3 protein, and CLN3 synthesized in a cell‐free environment act as prenylation substrates. Prenylation of GST‐CLN3T greatly enhances its association with membranes. Since prenylation occurs at protein termini, this modification at the C‐terminus of CLN3 may create an additional, terminal loop, which contradicts the assumption that the C‐terminus is free‐floating in the cytosol (Kaczmarski et al., 1999). Substitution of C435 by C435S does not affect CLN3 exit from the endoplasmic reticulum (ER) or transport to lysosomes in COS7 cells but trafficking rate and sorting efficiency are affected (Storch et al., 2007). Incubation with increasing concentrations of farnesyltransferase inhibitor L‐744,832 prevented prenylation of CLN3, which resulted in an increase in the fraction of CLN3 at the plasma membrane, suggesting that C‐terminal lipid modification of CLN3 is important for proper sorting (Storch et al., 2007). It is important to note that while sequence similarities and short‐term in vitro experiments are helpful, no experimental data exists demonstrating the function of PTMs of CLN3 protein in vivo.

### 2.7 Biosynthesis, trafficking, and intracellular localization of CLN3

In summary, CLN3 contains a farnesylation site at residues that are presumed to anchor the protein to intracellular or plasma membranes. However, mutagenesis of the putative farnesylation motif did not alter lysosomal localization of untagged, overexpressed CLN3. Thus, predicted farnesylation of CLN3 is not required for its lysosomal localization (Haskell et al., 2000; Pullarkat & Morris, 1997) but may have other, yet unidentified, roles.

#### 2.7.1 Biosynthesis, trafficking, and intracellular localization of wild‐type CLN3

Due to its low expression, hydrophobic nature, and lack of suitable antibodies capable of detecting endogenous CLN3, examination of the biosynthesis, PTMs, intracellular trafficking and localization of CLN3 were performed via overexpression in COS‐1, HeLa, baby hamster kidney (BHK), and normal rat kidney epithelia (NRK) cell lines. Most of the results are based on antibodies raised against the N‐terminal domain (h345, aa4‐19) or the large second cytosolic loop of human CLN3 (h385, aa242‐258, Q438, aa251‐265) (Haskell et al., 2000; Järvelä, Lehtovirta, Tikkanen, Kyttälä, & Jalanko, 1999).

Pulse‐chase experiments in transfected COS‐1 cells indicated that CLN3 is synthesized as an N‐glycosylated single‐chain polypeptide and is localized to the lysosomal compartment (Järvelä et al., 1998). Moreover, double immunoflourescence analyses in CLN3 overexpressing HeLa cells revealed strong co‐localization of CLN3 with the lysosomal marker protein Lamp1 (Kyttälä et al., 2004). This study likewise revealed a weak co‐localization of CLN3 with markers of the ER and early endosomes (early endosomal antigen 1; EEA1), whereas no colocalization was detected with the 300 kDa mannose 6 phosphate (M6P) markers of the *trans*‐Golgi/late endosome network, plasma membrane or mitochondria. Colocalization of CLN3 with lysosomal marker protein Lamp‐1 was also confirmed in transiently transfected BHK cells (Järvelä et al., 1999) and in neuronal cells. In transfected primary hippocampal neurons and glia cells CLN3 co‐localized mainly with the lysosomal marker Lamp‐1 and occasionally with EEA1 (Kyttälä et al., 2004). In transfected mouse primary telencephalic neurons, the distribution of CLN3 overlapped with lysosomal markers and synaptic vesicle marker SV2 (Järvelä et al., 1999). Indeed, the vast majority of studies on CLN3 have been in relation to the lysosome or have assumed that the primary function of CLN3 is lysosomal. However, it is important to note that CLN3 is present in multiple compartments of the cell, the significance of which is unknown. For instance, CLN3 was also detected at the Golgi/trans‐Golgi network and in more peripheral transport vesicles. Some particles were also detected at the plasma membrane (Kyttälä et al., 2004).

To rule out mislocalization of CLN3 due to high overexpression, lysosomal localization of CLN3 was confirmed by double immunoflourescence microscopy in NRK cells stably expressing low levels of CLN3 in an inducible manner (Girotti & Banting, 1996; Kyttälä et al., 2004; Luiro et al., 2004; Reaves & Banting, 1994). Moreover, cryoimmunoelectron microscopy of NRK cells stably transfected with untagged CLN3 demonstrated co‐localization of CLN3 with cathepsin D and LIMPII in lysosomal structures.

##### Lysosomal sorting motifs of CLN3

**Figure 3:**
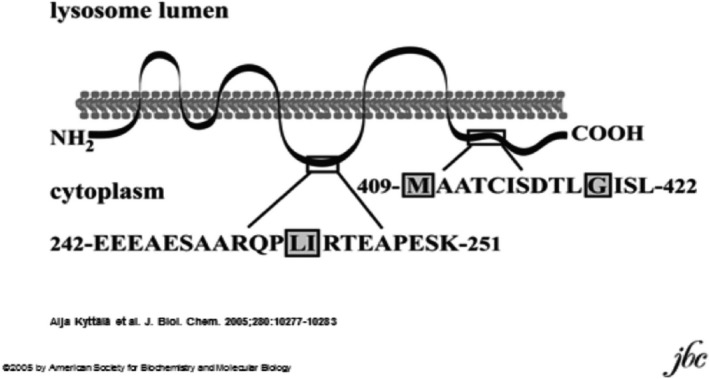
Lysosomal targeting motifs of CLN3

Traditionally, M6P‐tagged lysosomal enzymes are transported to late endosomes via vesicular transport. To test whether CLN3 is transported by the same mechanism as lysosomal enzymes, investigators expressed GFP‐CLN3 in CHO cells in presence of I‐M6P. No radioactive signal was incorporated into GFP‐CLN3 suggesting that CLN3 is directed to the lysosomal membrane by an alternative mechanism (Michalewski et al., 1999). Indeed, lysosomal targeting of membrane proteins is mediated by short linear sequences located in their cytosolic domains (Braulke & Bonifacino, 2009). These include tyrosine and acidic cluster dileucine‐based lysosomal sorting motifs which fit the consensus sequences (YXXO) and (D/E)XXXL(L/I), respectively, where X can be any amino acid and O is an amino acid with a large hydrophobic side chain (Bonifacino & Traub, 2003). These sorting motifs interact with cytosolic heterotetrameric adapter proteins AP1‐5 which mediate the packaging of transmembrane cargo into vesicles (Robinson, 2015). Amino acid sequence analysis of the carboxy‐terminal region of CLN3 suggests that the C‐terminal contains one or more tyrosine‐binding motifs (370–374, 378–382, 387–391) which are linked to cytoplasmic adapter complexes involved in sorting of integral membrane proteins to lysosomes (Höning, Sandoval, & von Figura, 1998; Ohno et al., 1998). CLN3 also possess a novel dominant dileucine‐based sorting signal in the predicted second cytoplasmic loop, EEE(X)8LI, and an unconventional *M*(X)9G sorting motif in the C‐terminal tail (Kyttälä et al., 2004; Storch et al., 2007). These complex sorting motifs are required for the transport of CLN3 to lysosomes. Using binding assays and immunoflourescence techniques, researchers determined that the dileucine motif binds both AP‐1 and AP‐3 in vitro and both adaptor complexes are required for sequential sorting of CLN3 protein (Kyttälä et al., 2005) (Figure 3).

##### Localization of endogenous CLN3 protein

Ezaki and coworkers demonstrated by subcellular fractionation of mouse livers that endogenous CLN3 is present in lysosomal fractions positive for the lysosomal markers cathepsin B, cathepsin D, Lamp1, Lamp2, and Limp2 (Ezaki et al., 2003). No codistribution of CLN3 with the mitochondrial marker subunit IV of cytochrome oxidase or the Golgi apparatus marker G58K was found in mouse liver. Immunohistochemistry of rat liver showed partial colocalization of endogenous CLN3 with the lysosomal marker acid phosphatase and the late endosomal marker lysobisphosphatidic acid. However, CLN3 did not overlap with EEA1, the *cis*‐Golgi marker protein GM 130, or the ER marker protein disulfide isomerase (PDI, Ezaki et al., 2003). Lysosomal localization of endogenous CLN3 was also demonstrate in human tissue by a proteomic approach using purified membranes of placental lysosomes (Schröder, Elsässer, Schmidt, & Hasilik, 2007).

##### Localization of CLN3 mutant protein

More than 60 different mutations in the *CLN3* gene have been identified in patients with CLN3 disease (see Table 1 and http://www.ucl.ac.uk/ncl/cln3.shtml). The most common genomic deletion (of 966 bp) results in the biosynthesis of a truncated CLN3 polypeptide composed of 153 canonical amino acids followed by 28 novel amino acids (Batten Disease Consortium, 1995). Based on experimentally determined membrane topology of the Ruonala group the mutant, truncated CLN3 protein is composed of the first two transmembrane domains and a large, C‐terminal cytosolic domain (Ratajczak et al., 2014). Based on an alternative schematic model of Nugent and coworkers the mutant, truncated CLN3 is composed of the first three transmembrane domains followed by 28 novel amino acids located on the luminal side of the membrane (Nugent et al., 2008). In both cases, mutant CLN3 lacks three or four transmembrane domains, the cytosolic loop, and the C‐terminal domain containing the lysosomal sorting motifs and the C‐terminal CQLS farnesylation site (Kyttälä et al., 2004; Storch et al., 2007).

Pulse‐chase analyses of truncated CLN3 expressed in BHK cells revealed a 24 kDa polypeptide which was not further processed in a 6 hr chase period (Järvelä et al., 1999). Double immunoflourescence analyses of truncated CLN3 expressed in BHK cells revealed major co‐localization of CLN3 with the ER marker PDI indicating its retention in the ER (Järvelä et al., 1999). In line with these findings, substitution of the entire C‐terminal domain of CLN3 with cytoplasmic tails of M6P receptors led to retention of chimeric proteins in the ER indicating the importance of the CLN3 C‐terminal for proper ER exit (Storch et al., 2007).

*It has long been a matter of debate whether patients that are homozygous for the 966bp deletion in CLN3, produce a biologically active mutant protein. Studies conducted by investigators at the University College London comparing healthy, and affected patient fibroblasts in the presence of RNA interference, support the presence of mutant CLN3 transcripts and postulate the presence of CLN3 protein activity* (Kitzmüller et al., 2008)*. In contrast, researchers at Sanford Children's Health Research Center found a substantial decrease in the transcript level of truncated CLN3 in patient fibroblasts, together with the analysis of transcripts expressed in the Cln3Dex1‐6 mouse and *in silico* prediction of the expected consequences of truncated protein, support the argument that nonsense‐mediated decay ensures that no functional (mutant) protein is made* (Chan, Mitchison, & Pearce, 2008; Miller, Chan, & Pearce, 2013)*. Moreover, researchers at Massachusetts General Hospital provide further evidence that support the role of nonsense‐mediated decay in the regulation of mutant CLN3 protein. However, Northern blot analyses of liver, kidney and brains of wild‐type and homozygous mutant Cln3Dex7/8 knock‐in mice revealed decreased yet stable levels of mutant RNA consistent with the presence of CLN3 mRNA in patient tissue (*Cotman et al., 2002 *; "Isolation of a novel gene underlying Batten disease, CLN3. The International Batten Disease Consortium,"* 1995 *)* *. Unfortunately, due to challenges associated with the ability of current reagents (antibodies) to detect endogenous CLN3 protein, no consistent results regarding intracellular localization or quantification of the protein have been possible for either wild‐type, mutant, or variant forms of CLN3 protein.*

Expression and localization studies of disease‐associated, *CLN3* missense mutations suggest that reduced or complete loss of CLN3 function results in decreased protein half‐life rather than a mislocalization. These studies also indicated that in CLN3 disease, caused by missense mutations, it is the loss of protein and not mislocalization that contributes to pathogenesis. In BHK cells expressing CLN3 E295K, the mutant CLN3 protein co‐localized with Lamp‐1 indicating correct lysosomal localization (Järvelä et al., 1999). Moreover, in transiently transfected human epithelial lung carcinoma cells (A‐549) mutant CLN3 with patient‐derived missense mutations V330F, R334H, L101P, L170P, and E295K colocalized with the lysosomal marker Lamp‐1, further supporting proper lysosomal localization with these mutations (Haskell et al., 2000). Conversely, expression of CLN3 carrying nonsense and frameshift mutations led to a retention of the protein in the ER. In HeLa cells transiently expressing p. Glu399X or p. CLN3 fsG424, mutant CLN3 colocalized with the ER protein PDI in immunoflourescence analyses indicating retention in the ER (Kyttälä et al., 2004). No experimental evidence exists on the consequences of other nonsense mutations (p.Trp35X, p.Glu17X, p.Glu72X, p.Arg89X, p.Ser161X, p.Ser162X, p.Tyr199X, p.Gln211X, p.Lys262X, p.Glu395X, p.Tyr322X, p.Gln327X, p.Gln352X, p.Thr400X, p.Ser423X) or frameshift mutations (p.Thr80Asn fsX12, p.Tyr124Leu fsX36, p.Arg127Pro fsx55, p.Arg127Gly fsX54, p.Gly154Ala fs29, p.Val142Leu fsX39, p.Gly187Asp fsX48, p.Gly190Glu fsX65, p.Ala196Gly fsX40, p.Ser208Phe fsX28, p.Gly264Val fsX29, p.His315Gln fsX67, p.Leu350Cys fsX27, p.Leu379Met fsX11, p.Leu425Ser fsX87) identified in CLN3 patients. For a visual representation of disease‐causing mutations, see Figure 2. For more and continually updated information regarding disease‐causing mutations please see http://www.ucl.ac.uk/ncl/cln3.shtml.

*Transient expression of the 966bp deletion, a common JNCL mutation and Q295K, a missense mutation predicted to be in the 5th transmembrane of the 6‐transmembrane model described by Nugent et al* (Nugent et al., 2008) *demonstrated that* CLN3 *protein with the common mutation is retained in the ER, whereas, Q295K mutants localize to the expected lysosomal compartment* (Järvelä et al., 1999)*. Q295K is associated with an atypical presentation of juvenile Batten disease. Visual failure initiates and proceeds similar to children with the common deletion. However, normal MRI results have been reported for 2 decades longer than in patients with the common deletion* (Järvelä et al., 1997; Wisniewski, Connell, et al., 1998)*.*

*NB: There is an error in Jarvela et al 1999 reading the amino acid code. The missense mutation is a change of glutamic acid (not glutamine) to lysine (i.e. E295K, not Q295K)*

*The 461–677 common deletion mutant localizes to the cell soma whereas wild‐type and Q295K co‐localize to the cell soma and neurites. The authors further report CLN3 co‐localizes with synaptic vesicle marker SV2 (antibody developed by Kathleen Buckley Harvard Medical School, Boston MA). Localization of wild‐type CLN3 protein to synaptic vesicles has not been confirmed by another laboratory. In 2001, Luiro and colleagues reported, using the same polyclonal antibody raised against amino acid residues (242–258, EEEAESAARQPLIRTEA), that CLN3 protein targets to synaptic fractions but not synaptic vesicles* (Järvelä et al., 1998; Luiro, Kopra, Lehtovirta, & Jalanko, 2001)*. Human retinal cells transfected with CLN3 and immunostained with an antibody raised against the CLN3 peptide 242–258 showed a beads‐on‐a‐string pattern in neurites, partial co‐localization with SV2, and no co‐localization with LAMP1* (Luiro et al., 2001)*.*

##### Localization of epitope‐tagged CLN3 protein

Golabek and colleagues reported results obtained from expressing full‐length CLN3 fused with GFP in COS‐1, HeLa, and human neuroblastoma (SK‐N‐SH) cell lines. Using western blotting, Percoll density gradient fractionation, and Triton X‐114 extraction, the authors demonstrated that the product of the *CLN3* gene is a highly glycosylated protein found within membrane‐enriched fractions (Golabek et al., 1999). [The authors state that the results of their experiments indicate that CLN3 protein is lysosomal. However, the fractionation methods used do not separate subcellular and plasma membranes from one another, therefore making it impossible to tease out the precise localization of CLN3].

Kida and colleagues expressed full‐length and truncated CLN3 fused to GFP at its N‐terminus (GFP‐CLN3) in CHO and SK‐N‐SH cell lines (Kida et al., 1999). Using co‐immunoflourescence analyses the authors showed that full‐length GFP‐CLN3 fusion protein colocalizes with lysosomal markers Lamp‐1 and Lamp‐2 and with the late endosomal marker Rab7. GFP‐CLN3 was found in the ER, in a few vesicular structures of the Golgi apparatus, and in COPI‐coated vesicles, most likely due to the presence of newly synthesized CLN3 trafficking from the ER to the Golgi apparatus. GFP‐CLN3 did not colocalize with markers of mitochondria or plasma membrane. In contrast, truncated GFP‐CLN3 that lacked either the C‐terminal domain (GFP‐CLN3 aa 1–322 and GFP‐CLN3 aa 1–138) or the N‐terminal domain (GFP‐CLN3 aa138–438) did not codistribute with lysosomal markers thus, indicating their mislocalization. Most of the truncated fusion proteins either localized to the cytoplasm, nucleus, or ER similar to GFP alone. However, mutant CLN3, with double‐point mutations Leu425Leu426 into Gly425Gly426, at its putative dileucine motif, localized to lysosomes, in a similar fashion to wild‐type, full‐length GFP‐CLN3 (Kida et al., 1999). Studies in CHO cells stably expressing GFP‐CLN3 in the presence of the pharmacological N*‐*glycosylation inhibitor tunicamycin suggest that N‐glycosylation is not required for correct targeting of CLN3 to lysosomes. However, treatment with Monensin, a Na+ ionophore which blocks glycoprotein secretion, produced retention of GFP‐CLN3 in vesicular structures of the Golgi apparatus in the perinuclear space, suggesting that CLN3 fusion protein is transported to the lysosomal compartments through the trans‐Golgi cisternae (Kida et al., 1999).

In contrast to the data described above, Haskell and coworkers showed a nonvesicular distribution of N‐terminal tagged GFP‐CLN3 which overlapped with the Golgi marker beta‐COP in transfected A549 cells, indicating its localization to the Golgi apparatus (Haskell, Derksen, & Davidson, 1999). Haskell et al also found no colocalization of GFP‐CLN3 with lysosomal marker Lamp‐1 or the mitochondrial marker mtHSP60. When disrupted in the presence of brefeldin A, ER‐like staining was noted. The authors postulated that, if wild‐type CLN3 protein localizes to lysosomes and mitochondria under normal conditions, their N‐terminal tag disrupts such localization.

CLN3 fused to GFP at its C terminus (CLN3‐GFP) mainly colocalized with Golgi markers as determined by immunofluorescence analysis (Kremmidiotis et al., 1999). In transiently transfected fibroblasts, HeLa and COS‐7 cells and stably transfected HeLa cells CLN3‐GFP fluorescence codistributed with wheat germ agglutinin coupled to Texas red. Stable expression of CLN3‐GFP in HeLa cells showed perinuclear, asymmetric localization with the Golgi apparatus, minor localization to the ER and lysosomes, and no apparent localization to the nucleus, mitochondria, or cell surface membrane. A juxtanuclear, asymmetric Golgi‐like localization pattern was also observed in transiently transfected HeLa, COS‐7, and fibroblast cells (Kremmidiotis et al., 1999).

#### 2.7.2 Overexpressed, mutant CLN3 protein

More than 80% of the GFP–CLN3 fusion protein can be extracted by phase separation in a solution of Triton X‐114 indicating that CLN3 is a highly hydrophobic membrane protein (Michalewski et al., 1999).

Using immunoelectron microscopy, investigators analyzed the intracellular processing and localization of two CLN3 protein mutants, 461–677 deletion, or the “1 kb” deletion present in 85% of CLN3 alleles (73% of affected patients) and E295K [corrected], a rare missense mutation. Pulse‐chase labeling and immunoprecipitation of the 461–677 deletion and E295K mutation indicated that 461–677 deletion protein is synthesized as a ~24 kDa polypeptide, whereas the maturation of E295K mutant [corrected] resembles wild‐type CLN3 protein. Transient expression of the two mutants in BHK cells showed that 461–677 deletion protein is retained in the ER, whereas E295K [corrected] mutant was capable of reaching the lysosomal compartment. Using mouse primary neurons, investigators showed that wild‐type and E295K [corrected] mutant CLN3 proteins localize to the soma and neurites, whereas the 461–677 deletion protein is not found in neurites (Järvelä et al., 1999).

## 3 TISSUE DISTRIBUTION

CLN3 is universally expressed in multiple human tissues. Immunoblot, immunohistochemistry, Northern blot, and PCR analyses reveal CLN3 protein and mRNA expression in the nervous system, glandular/secretory system, skeletal muscle, gastrointestinal tract, and cancer tissues (Chattopadhyay & Pearce, 2000; Margraf et al., 1999; Persaud‐Sawin, McNamara, Rylova, Vandongen, & Boustany, 2004; Rylova et al., 2002).

In the brain, reactivity for CLN3 is present in astrocytes and neurons, and is more pronounced in the cells of the gray matter, where a larger percentage of astrocytic cells were stained. Capillary endothelium also showed cytoplasmic CLN3 expression. Overall, the expression is similar in intensity and distribution in all of the areas of brain examined, including frontal and temporal cerebral lobes, hippocampus, basal ganglia, and pons (Chattopadhyay & Pearce, 2000; Margraf et al., 1999). Peripheral nerves also express CLN3 (Margraf et al., 1999; Persaud‐Sawin et al., 2004).

In the glandular/ secretory system CLN3 is present in the pancreas (islet somatostatin‐secreting delta cells) (Boriack & Bennett, 2001; Margraf et al., 1999), kidney, testis (in the Sertoli and maturing germ cells), lungs, lymph nodes, placenta, uterus, prostate, ovary, liver, adrenal gland, thyroid, salivary gland, and mammary gland (Chattopadhyay & Pearce, 2000; Margraf et al., 1999).

In the gastrointestinal tract, CLN3 expression is found in stomach, duodenum, jejunam, ileum, ileocecum, appendix, colon and rectum (Chattopadhyay & Pearce, 2000; Rylova et al., 2002). CLN3 is also expressed in fibroblasts (Persaud‐Sawin et al., 2004), heart, and skeletal muscle (Chattopadhyay & Pearce, 2000).

In cancer tissues, CLN3 mRNA and protein are overexpressed in glioblastoma (U‐373G and T98g), neuroblastoma (IMR‐32, SH‐SY5Y, and SK‐N‐MC), prostate (Du145, PC‐3, and LNCaP), ovarian (SK‐OV‐3, SW626, and PA‐1), breast (BT‐20, BT‐549, and BT‐474), and colon (SW1116, SW480, and HCT 116) cancer cell lines, but not in pancreatic (CAPAN and As‐PC‐1) or lung (A‐549 and NCI‐H520) cancer cell lines. Indeed, CLN3 expression is 22%–330% higher in 8 of 10 solid colon tumors when compared with the corresponding normal colon tissue control (anHaack et al., 2011; Rylova et al., 2002; Zhu et al., 2014).

### 3.1 Gene expression data analysis for CLN3

Tissue expression of CLN3 was retrieved from several sets of expression data collected across the ArrayExpress database (Rustici et al., 2013) and NCBI GEO (GSE1133, GSE2361, GSE7307, GSE30611) (Barrett et al., 2013) (Table 6).

**Table 6:** NCBI GEO data sets used for tissue specific expression analysis

Figure (below)	Dataset	Species	Platform	Description
Figure 4	E‐MTAB‐62	Human	Affymetrix HG‐U133A	Human gene expression atlas of 5,372 samples representing 369 different cell and tissue types, disease states and cell lines.
Figure 5	GSE10246	Mouse	Affymetrix Mouse Genome 430 2.0 Array	Multiple tissues were taken from 182 naïve male C57BL6 mice and hybridized to mouse genome arrays to profile a range of gene expressions in normal tissues.
Figure 6	GSE53960	Rat	Illumina HiSeq 2000	As part of the SEQC consortium efforts, a comprehensive rat transcriptomic BodyMap created by performing RNA Seq on 320 samples from 11 organs of both sexes of juvenile, adolescent, adult and aged Fischer 344 rats.
Figure 7	GSE1133	Human	Affymetrix HG‐U133A, GNF1M (non‐commercial), GNF1H (non‐commercial)	Custom arrays that interrogate the expression of the vast majority of protein‐encoding human genes were developed and used to profile a panel of 79 human tissues. The resulting data set provides the expression pattern for thousands of predicted genes, as well as known and poorly characterized genes.
Figure 8	GSE7307	Human	Affymetrix HG‐U133 Plus 2	677 samples representing 90 distinct tissues from normal and diseased human tissues were profiled for gene expression using the Affymetrix U133 plus 2.0 array

Briefly, each Affymetrix microarray data set was downloaded and preprocessed by MAS5.0 algorithm followed by quantile normalization. Updated Entrez‐centric BrainArray CDF files were used for normalization. RNA‐Seq datasets were downloaded in the already pre‐processed formats from the ReCount and SEQC ("Rat Body Map") websites, respectively. In each dataset, gene expression levels were transformed into Z‐scores by gene centering on zero and dividing the centered profiles by their standard deviation. The box plot for each tissue reflects the distribution of probe set expression signals (log2‐scaled) across the samples of this tissue. The median signal is depicted as black line in the middle of the box; box borders represent the 25th and 75th percentiles of signal distribution. Empty dots represent the “outliers” – samples with unusually high or low expression signal.

**Figure 4:**
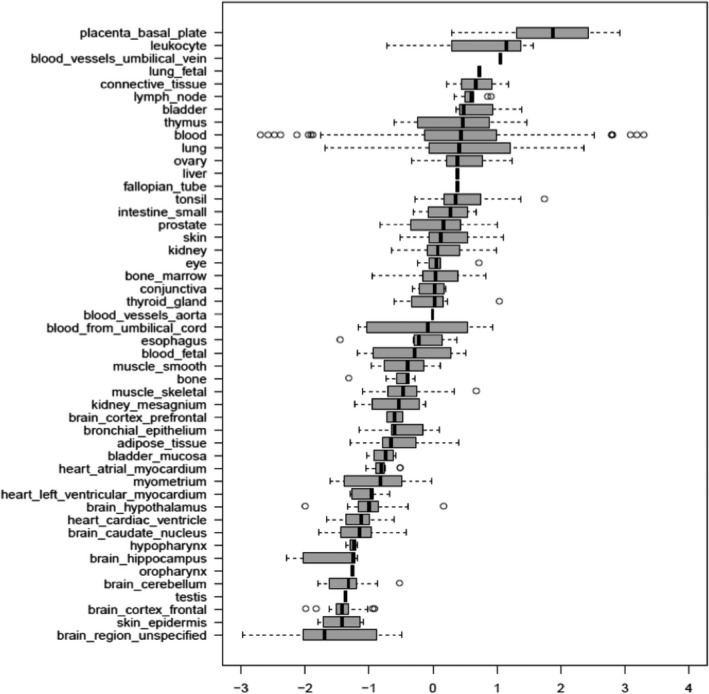
Tissue expression profile of CLN3 across dataset E‐MTAB‐62 (human)NCBI GEO GSE2361

**Figure 5:**
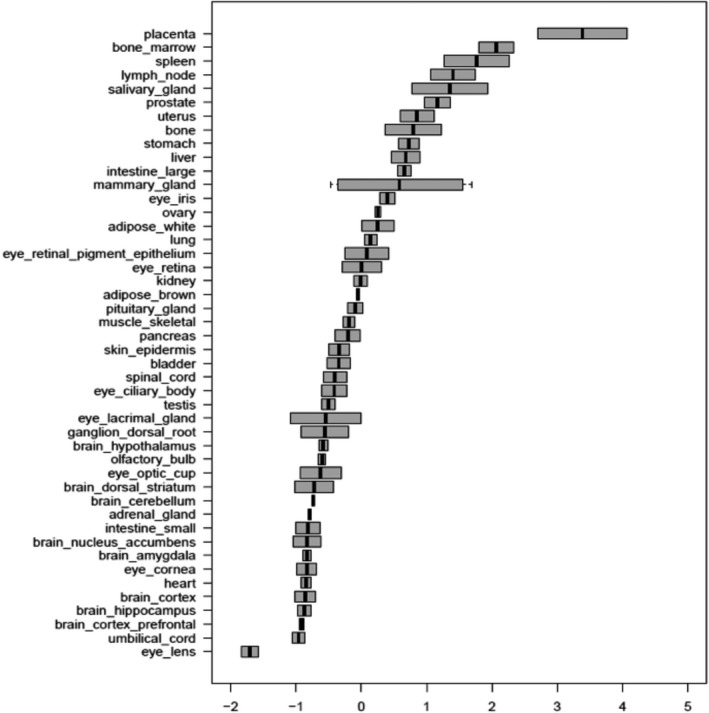
Tissue expression of CLN3 across NCBI GEO data set GSE10246 (mouse)

**Figure 6:**
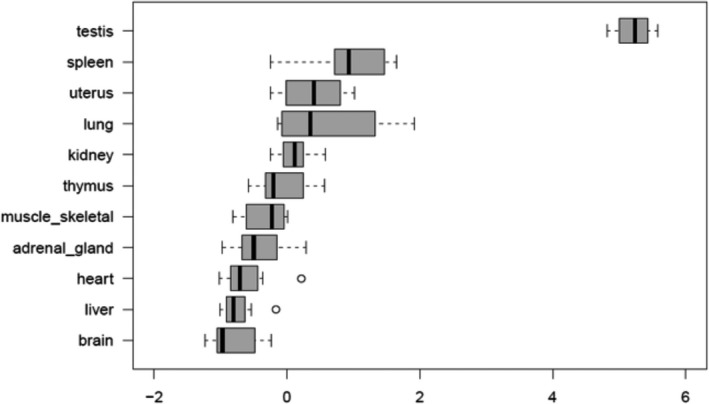
Tissue expression of CLN3 across NCBI GEO data set GSE5396 (rat)

**Figure 7:**
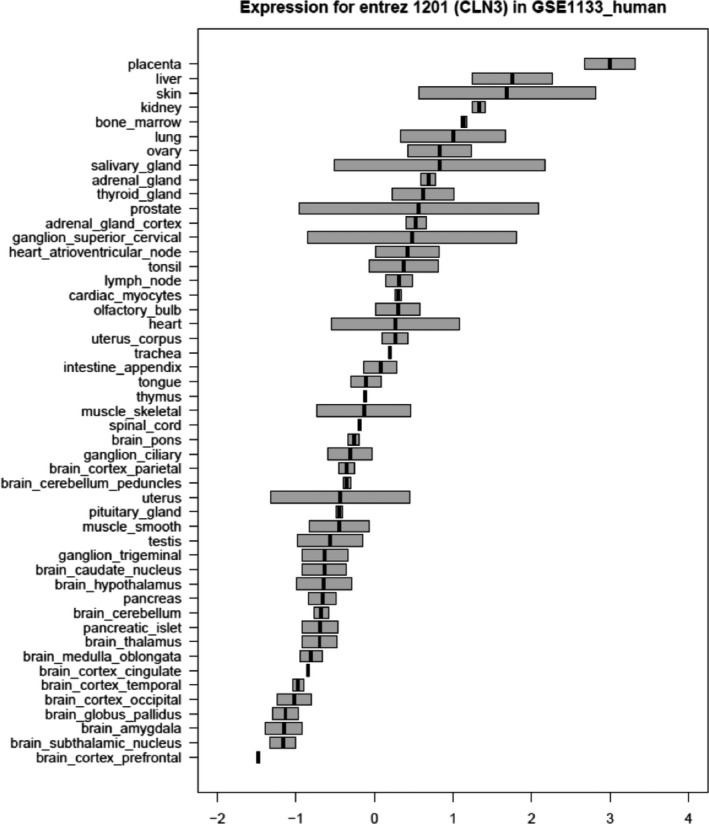
Expression of CLN3 in human tissues according to the NCBI GEO GSE1133 data set

**Figure 8:**
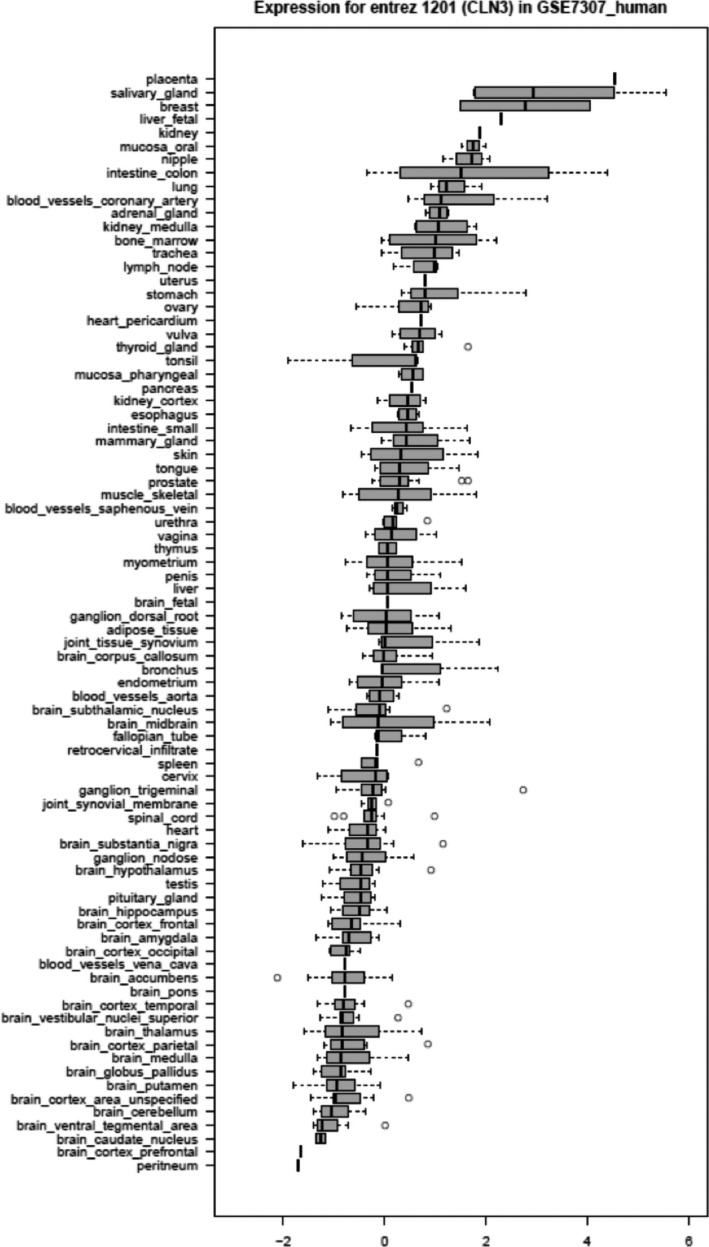
Expression of CLN3 in human tissues according to the NCBI GEO GSE7307 data set

The expression of human CLN3 across the E‐MTAB‐62 dataset is plotted in Figure 4, while the mouse GSE10246 and rat GSE53960 data sets are presented in Figure 5 and Figure 6 respectively. We see the highest CLN3 expression in human placenta and leukocytes. Similarly, CLN3 expression is highest in mouse placenta and leukocyte‐associated tissues (bone marrow, spleen, and bone marrow). The tissue distribution available in the rat dataset is limited but the CLN3 expression profile differs noticeably, in that expression is highest in rat testis and then spleen, while testis expression in human is relatively low (~ −1.5 relative to median tissue expression). Additional human tissue distribution plots of each dataset are available in Figures 7 and 8.

Expression of CLN3 across human tissues and cell types was independently assessed using large‐scale data: such as public microarray data sets. The most widely used data set for whole‐genome human tissue expression is GenAtlas; with 79 human tissues examined by Affymetrix HG U133A microarrays normalized using the GCRMA algorithm. Most tissue samples have only 2 replicates, so the conclusions about expression specificity must be drawn with caution (Figure 7).

**Figure 9:**
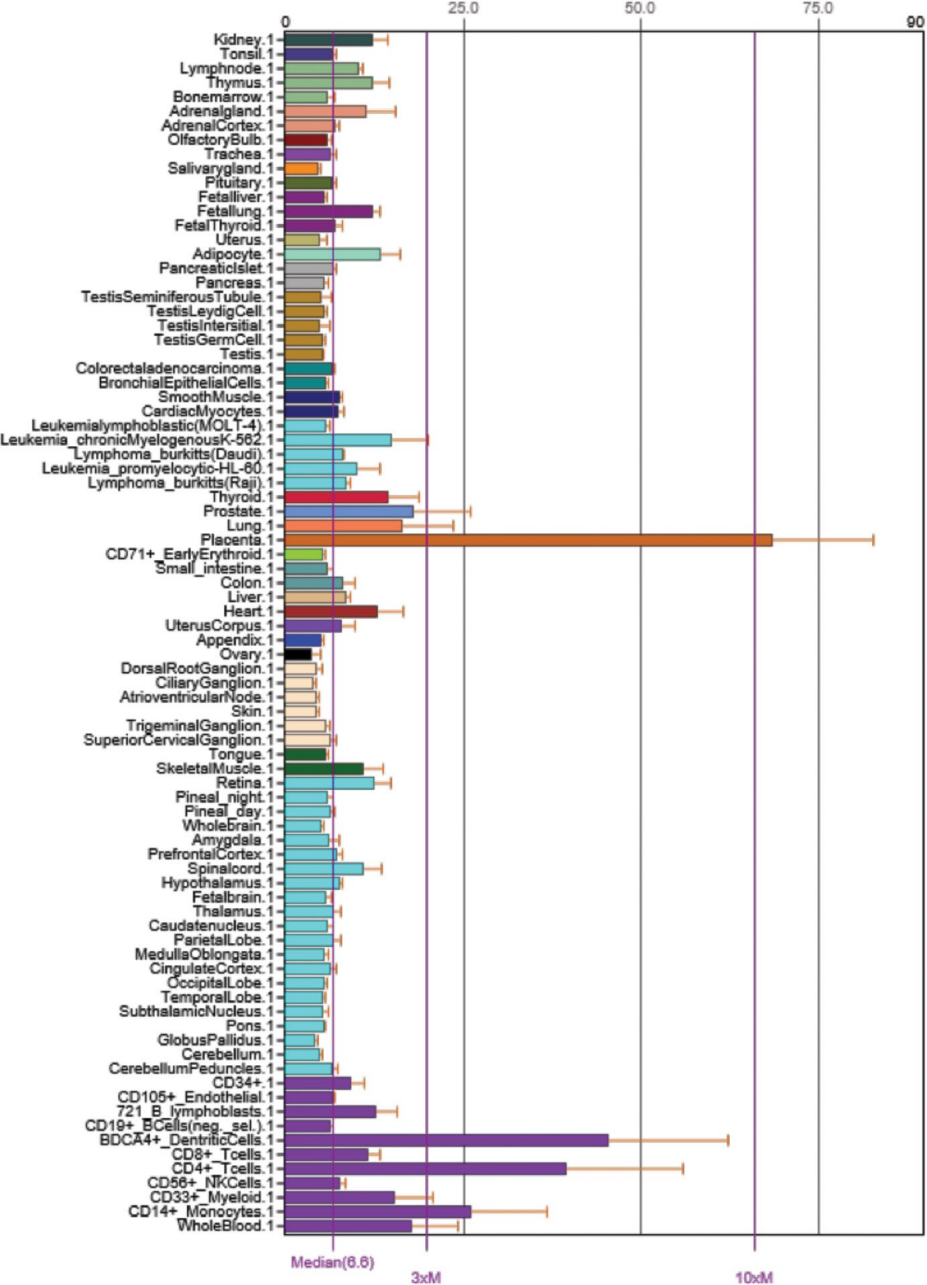
Expression of CLN3 in human tissues according to the Gene Atlas data set (Su et al., 2004; Wu et al., 2009)

Expression of CLN3 is uniform across the majority of tissues profiled in the GeneAtlas data set (Figure 9). The first (dashed) line indicates mean signal of 9.5 RFU. A few tissues have elevated signal of 3X mean (BDCA4 + Dendritic cells, CD4 + helper T‐Cells) and maximum expression is seen in placenta.

**Figure 10:**
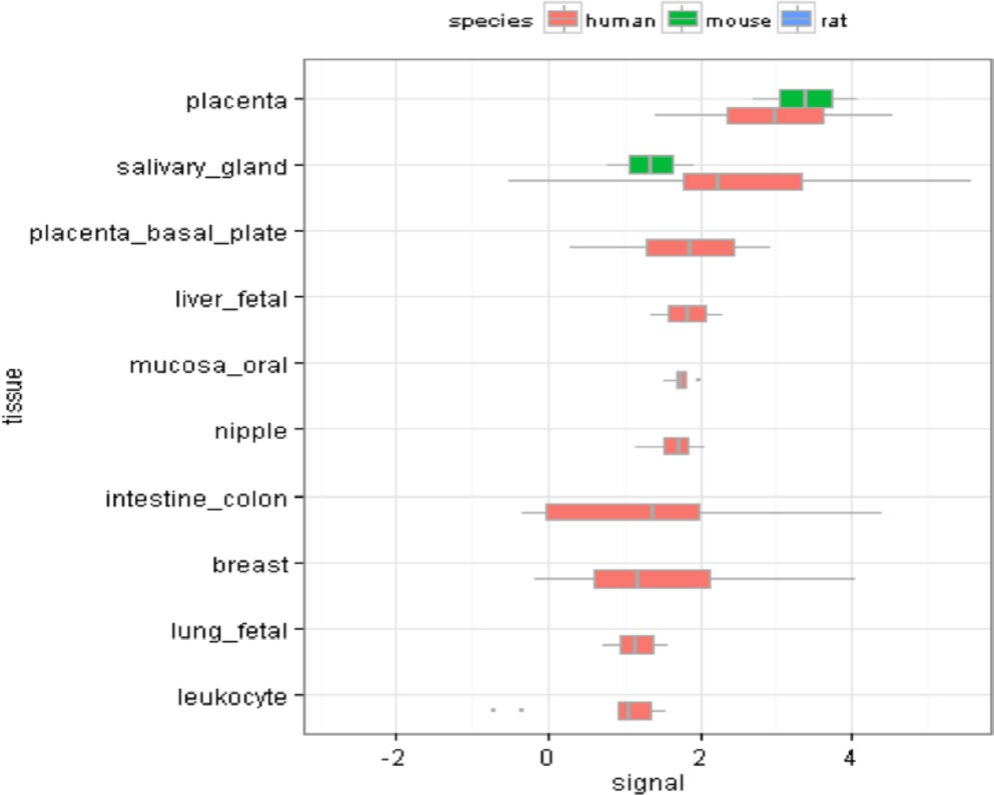
Cross species tissue expression for top ten tissues by expression level in human

When compared across species, the shared tissue samples show a clear trend towards high expression in placenta (Figure 10).

#### 3.1.1 CLN3 RNA expression data using GTex Portal

Quantification of gene expression in this data set is mainly done using RNA‐seq. Tissue‐specific gene expression data can be found at http://www.gtexportal.org/home/gene/CLN3 (Lonsdale et al., 2013). CLN3 seems to be highly expressed in colon as compared to other tissues in this dataset. This data does not include placenta and hence, cannot be compared to the above microarray datasets.

#### 3.1.2 Protein expression data analysis for CLN3

The largest proteomics database on tissue expression, Protein Atlas (Uhlen et al., 2010), also confirms widespread expression of CLN3 throughout the organism. Unfortunately, no immunohistochemistry or western blot analysis are available for CLN3 to determine the range or intensity of protein distribution across tissues. This is most likely due to the lack of high‐quality antibodies to CLN3 protein. To date, there are over 30 antibodies in the academic and pharmaceutical sectors. All exhibit either limited CLN3 reactivity, high nonspecific binding or both.

#### 3.1.3 Tissue distribution of CLN3 protein in mice

Using two CLN3‐ specific antibodies, one directed at the N‐terminal (5–19) and the other at the mid‐region (225–280), CLN3 was detected in various mouse tissues (Ezaki et al., 2003). Brain, liver, pancreas, and spleen were tested using both the antibodies. Mouse CLN3 protein was detected as a smear between 45 and 66 kDa in liver, kidney, pancreas, and spleen. In the brain, a CLN3 band is observed closer to 55 kDa. With both antibodies CLN3 signal in brain tissue was weaker than in the other tissues mentioned above.

## 4 PROTEIN‐PROTEIN INTERACTIONS

This section presents protein‐protein and nucleic acid‐protein interactions of CLN3. The interactions contain directionality, effect (activation, inhibition, etc.) and mechanism (binding, phosphorylation, transcriptional regulation, etc.).

As stated previously, the function of the CLN3 has not been completely elucidated. This is not surprising as transmembrane proteins, like CLN3, are highly hydrophobic and difficult to stabilize in solutions for study. This section is designed to answer specific questions regarding binding of CLN3 to other proteins. Some statements mention putative functions for CLN3. For a full exploration of the proposed functions, the reader should consult the primary literature.

### 4.1 Interactions with Kinases and Phosphatases

As mentioned in the phosphorylation section above, CLN3 has been demonstrated to interact with, and be phosphorylated by, PKA, PKG, and casein kinase II. Moreover, it is dephosphorylated by protein phosphatase 1 or protein phosphatase 2A (Michalewski et al., 1998, 1999) see phosphorylation section for more information. The phosphorylation of CLN3 may play a role in its interaction with membrane compartments, regulation of protein interactions and formation of functional complexes.

### 4.2 C2‐Ceramide

The genes that are transcriptionally regulated during ceramide‐mediated cell death are poorly understood. Puranam et al. found that CLN3 does not inhibit C2‐ceramide‐induced apoptosis but modulates endogenous ceramide synthesis and suppresses apoptosis by preventing the generation of ceramide (Puranam, Guo, Qian, Nikbakht, & Boustany, 1999). Accordingly, overexpression of CLN3 protects cells from vincristine‐, staurosporine‐, and topside‐induced apoptosis, but not from ceramide‐induced cell death (Puranam et al., 1999). In addition, C2‐ceramide has been found to induce the expression of CLN3 in PC12 cells, which could represent a negative feedback mechanism regulating endogenous ceramide generation for cellular protection from cell death (Decraene et al., 2002).

### 4.3 Interactions with Endosomal‐Lysosomal proteins

Although the function of CLN3 protein remains unknown, several findings support the conclusion that lysosomes and endosomes are prominent sites of CLN3 activity. CLN3 contains multiple lysosomal targeting signals including a non‐conventional signal in the C‐terminus, and although any of these are sufficient for transport of CLN3 to lysosomes, all are required for optimal transport efficiency (Kyttälä et al., 2004). CLN3 binds directly to active, guanosine triphosphate (GTP)‐bound Rab7 and Rab‐interacting lysosomal protein as confirmed by mammalian two‐hybrid experiments with a peptide corresponding to amino acids 1–40 of CLN3. These experiments suggest that CLN3 binds to Rab7 via its N‐terminus and that this interaction occurs most favorably with the GTP‐bound form of Rab7 (Kyttälä et al., 2004; Uusi‐Rauva et al., 2012). Rab7 facilitates vesicular transport and delivery from early to late endosomes and late endosomes to lysosomes. The role of Rab7 in vesicular transport is dependent on its interactions with effector proteins, among them RILP, which aids in the recruitment of active Rab7 (GTP‐bound) onto dynein‐dynactin motor complexes to facilitate late endosomal transport on the cytoskeleton (Agola et al., 2015).

To examine putative interactions between CLN3 and microtubule‐binding protein, Hook1, investigators conducted in vitro binding assays with cytoplasmic Hook1 and two putative cytoplasmic domains of CLN3 (1–33 and 232–280). When compared with the GST vector alone, putative CLN3 cytoplasmic domains (1–33 and 232–280) bound with low affinity to Hook1 (Luiro et al., 2004). When Hook1 and CLN3 proteins were co‐expressed with individual GFP‐tagged Rab 7, 9, and 11, Hook1 was found to specifically interact with Rab7, Rab9, and Rab11. In contrast no direct interactions between CLN3 and the Rab proteins were found (Luiro et al., 2004). These findings implicate CLN3 in a complex cellular machinery connecting cytoskeletal dynamics to endocytic membrane trafficking. It is proposed that this interaction may be disturbed in the absence of CLN3, thus leading to endocytic dysfunction in CLN3 deficient cells (Luiro et al., 2004). Indeed wild‐type CLN3 protein (CLN3p, 48–52 kD) traffics from Golgi to lipid rafts at the plasma membrane *via* Rab4‐ and Rab11‐positive endosomes (Persaud‐Sawin et al., 2004). However, mutant CLN3 protein does not appear to localize to the plasma membrane in JNCL fibroblasts. Instead, mutant CLN3p was retained within the Golgi and partially mis‐localized to lysosomes, failing to reach recycling endosomes, plasma membrane, or lipid rafts (Persaud‐Sawin et al., 2004). Moreover, the yeast homolog of CLN3, BTN1, has also been shown to play a role in endosome‐Golgi retrograde transport by regulating SNARE protein function (Kama et al., 2011). Although BTN1 does not directly interact with SNAREs, it was shown to modulate Sed5 phosphorylation by regulating Yck3, a palmitoylated endosomal kinase. This may involve modification of the Yck3 lipid anchor, as substitution with a transmembrane domain suppresses the deletion of BTN1 and restores trafficking (Kama et al., 2011).

### 4.4 Interactions with Calsenilin/dream/KChIP3

Yeast two hybrid (Y2H) and immunoprecipitation assays show that Calsenilin (also known as downstream regulatory element antagonist modulator (DREAM) and K+ channel interacting protein 3 (KChIP3)), a neuronal Ca^2+^‐binding protein, interacts with the C‐terminal region of CLN3 (385–438) and that an increase in Ca^2+^ concentration promotes the dissociation of CLN3 from calsenilin. Calsenilin has been found to act as a transcriptional repressor, and its activity has been linked with neuronal excitation and repolarization of K+ channel (An et al., 2000). Calsenilin binds DNA in a Ca^2+^ dependent manner and increases the expression of wild‐type or C‐terminal CLN3 and suppresses thapsigargin mediated cell death. Thapsigargin is a sarco/endoplasmic reticulum Ca^2+^‐ATPase pump inhibitor, a research tool used to raise cytosolic Ca^2+^ and induce Ca^2+^‐mediated cell death. In the absence of CLN3, such as in the case of CLN3 knockout mice or human SH‐SY5Y cells deficient in CLN3, cells are more sensitive to thapsigargin (Chang et al., 2007). The CLN3‐Calsenilin interaction was not confirmed by Tandem Affinity Purification coupled to Mass Spectrometry (TAM‐MS) combined with Significance Analysis of Interactome (SAINT) in human SH‐SY5Y cells (SH‐SY5Y‐NTAP‐CLN3, (Scifo et al., 2013)). More work is needed to understand the physical properties and functional role(s) of CLN3 and Calsenilin interactions.

In 2015, investigators utilized an autophagy assay, a process previously shown to be disrupted in the Cb*Cln3* ^Δ^ *^ex7/8/Δex7/8^* mouse model of the disease (Cao et al., 2006), which used green fluorescent protein‐tagged LC3 transgene to label autophagosomes in mouse cerebellar Cb*Cln3* ^Δ^ *^ex7/8/^* ^Δ^ *^ex7/8^* cell lines. Using these cell lines, investigators screened small molecule modifiers of autophagy to discover the sensitivity of disease cell models to alterations in autophagy which impact Ca^2+^ regulation. In these experiments, thapsigargin reproducibly displayed significantly more activity in mouse knock‐in cerebellar neurons as well as in induced pluripotent stem cells derived from patients with the common deletion. The mechanism of thapsigargin sensitivity was Ca^2+^‐mediated, and autophagosome accumulation in JNCL cells could be reversed by cytosolic Ca^2+^ chelation. Interrogation of intracellular Ca^2+^ handling highlighted alterations in ER, mitochondrial, and lysosomal Ca^2+^ pools and in store‐operated Ca^2+^ uptake (Chandrachud et al., 2015).

### 4.5 Interactions with other NCL proteins

It has long been presumed, due to the similarity of clinical features and pathological hallmarks between various NCLs, that NCL proteins are part of the same or similar cellular pathways and that there is some degree of interaction between NCL proteins. Some studies support this conclusion. The 13 proteins encoded by NCL genes do not all localize to endosomal/lysosomal pathways, some are situated in compartments of the secretory system such as the ER; these can be found in Table 7.

**Table 7:** CLN3 interactions with other NCLs

Gene	Gene Product (protein)	Protein description	Primary and Neuronal Location(s)	References
*CLN1*	Palmitoyl protein thioesterase 1, PPT1	soluble enzyme	Lysosomal	Persaud‐Sawin et al. (2007)
*CLN2*	Tripeptidyl peptidase 1, TPP1	soluble enzyme	Lysosomal	Vesa et al. (2002) and Persaud‐Sawin et al. (2007)
*CLN3*	CLN3 transmembrane protein	transmembrane protein	Late endosomal/Lysosomal, synaptosomes, axons	Storch et al. (2007)
*CLN4/DNAJC5*	Cysteine string proteinα	secretory vesicle protein		
*CLN5*	Ceroid‐lipofuscinosis neuronal protein 5	soluble (non) enzyme glycoprotein	ER, Lysosomal, neurites	Lyly et al. (2009)
*CLN6*	Ceroid‐lipofuscinosis neuronal protein 6	transmembrane protein	Endoplasmic Reticulum	Persaud‐Sawin et al. (2007)
*CLN7*	Major facilitator superfamily domain‐containing protein 8	transmembrane protein, endolysosomal transporter	Late endosomal/Lysosomal	
*CLN8*	unknown transmembrane protein, ER, ER‐Golgi intermediate complex	transmembrane protein	Endoplasmic Reticulum, ER‐Golgi intermediate complex	Persaud‐Sawin et al. (2007)
*CLN10/CTSD*	Cathepsin D	soluble lysosomal enzyme	Lysosomal	
*CLN11/GRN*	Progranulin	non enzyme; poorly understood		
*CLN12/ATP13A2*	P‐type ATPase	non enzyme; poorly understood		
*CLN13*	Cathepsin F	soluble lysosomal enzyme		
CLN14/KCTD7	Potassium channel tetramerization domain‐containing protein 7	probable transmembrane protein voltage‐gated potassium channel complex		

#### 4.5.1 Interaction with CLN1

Palmitoyl protein thioesterase 1 (PPT‐1), encoded by *ceroid‐lipofuscinosis, neuronal 1 (CLN1)*, is a small soluble glycoprotein involved in the catabolism of lipid‐modified proteins during lysosomal degradation. The encoded enzyme removes thioester‐linked fatty acyl groups such as palmitate from cysteine residues on multiple protein targets (Cho & Dawson, 1998; Cho, Dawson, & Dawson, 2000). Defects in this gene are linked to a rapidly progressing lysosomal disease by the same name, CLN1 disease. CLN1 disease may also be referred to as infantile neuronal ceroid lipofuscinosis (INCL) as the vast majority of reported cases of the disease present around 18 months of age. Like most pediatric forms of NCL, patients experience progressive vision loss, cognitive and motor deficits, seizures and early death. With the advent of increased genetic testing, cases of late infantile, juvenile and adult‐onset CLN1 have been reported suggesting that less severe mutations of the gene produce some or modified versions of the PPT‐1 protein (Van Diggelen et al., 2001). The possibility that CLN1 and CLN3 gene products interact with one another was raised by studies demonstrating that PPT‐1 localizes to synaptic vesicles and CLN3 to synaptosomes (Ahtiainen, Diggelen, Jalanko, & Kopra, 2003; Hellsten, Vesa, Olkkonen, Jalanko, & Peltonen, 1996; Lehtovirta et al., 2001; Luiro et al., 2001; Sleat et al., 1997). However, direct interaction between PPT1 and CLN3 protein was not demonstrated by co‐immunoprecipitation, while parallel studies with CLN1 and CLN2 demonstrated interaction between the two. In the same study no benefit to cellular growth or apoptosis was observed in CLN3 deficient cells transfected with CLN1, whereas benefits were observed with CLN2 and CLN6 expression (Persaud‐Sawin et al., 2007). More recent studies using TAM‐MS combined with bioinformatics SAINT demonstrated an interaction between CLN3 and PPT1; however, it is not clear whether this interaction is direct (Scifo et al., 2013).

#### 4.5.2 Interaction with CLN2

The *ceroid‐lipofuscinosis, neuronal 2 (CLN2)* gene encodes tripeptidyl peptidase I (TPP‐1), a serine protease which cleaves N‐terminal tripeptides from the free N‐termini of small polypeptides and also shows minor endoprotease activity (Golabek et al., 2003, 2004). Mutations in CLN2 result in late‐infantile neuronal ceroid lipofuscinosis previously referred to as LINCL, but now more commonly known as CLN2. Normal human lymphoblasts and COS‐7 cell lysates immunoprecipitated with an anti‐CLN3 antibody and probed with an anti‐CLN2 antibody, detected a 48–50 kD band. This suggested that CLN2 and CLN3 physically interact with one another, which was further supported by co‐localization experiments performed in the same study (Persaud‐Sawin et al., 2007). In addition, C57BL/6 mice homozygous for targeted disruption of the *CLN3* gene exhibit elevated CLN2/TPP1 protease activity in the brain, implying a biochemical connection between the gene products of *CLN3* and *CLN2* (Mitchison et al., 1999). More recent studies using TAM‐MS combined with bioinformatics SAINT also demonstrated an interaction between CLN3 and TPP‐1; however, it is not clear whether this interaction is direct (Scifo et al., 2013).

#### 4.5.3 Dimerization of CLN3

A transmembrane topology where CLN3 contains 6 transmembrane domains with both the N‐ and C‐terminal domains facing the cytosol is currently favored and is supported by both computer modeling and experimental evidence (Kyttälä et al., 2004; Nugent et al., 2008; Ratajczak et al., 2014; Storch et al., 2007). An alternative model which predicts a 5‐transmembrane topology of CLN3 also exists (Mao, Foster, et al., 2003). However, neither the function of CLN3 nor its functional tertiary structure have been solved yet. When COS7 cells overexpressing N‐terminally Myc‐tagged CLN3 are permeabilized and incubated in the absence or presence of chemical cross‐linkers BS^3^ and DMS, tagged CLN3 forms SDS‐stable 88‐kDa proteins, presumed to correspond to a CLN3 homodimer (Storch et al., 2007). However, experimental artifacts may result in the formation of dimers or oligomers simply via hydrophobic interactions therefore, whether CLN3 forms a functional dimer remains to be confirmed.

#### 4.5.4 Interaction with CLN5

CLN5 protein (CLN5p) is a highly glycosylated protein of unknown function which, similar to CLN3, localizes to lysosomes and neurites (Holmberg et al., 2004; Isosomppi, Vesa, Jalanko, & Peltonen, 2002; Jalanko, Patrakka, Tryggvason, & Holmberg, 2001). Pathogenic mutations lead to its retention in ER/ Golgi and the Finnish variant late infantile form of NCL (vLINCLFin, (Holmberg et al., 2000; Savukoski et al., 1998). Late infantile, juvenile, and adult‐onset forms of CLN5 disease have been reported. Co‐immunoprecipitation and in vitro binding assays revealed that CLN3 protein interacts directly with wild‐type CLN5 synthesized as 47‐, 44‐, 41‐, and 39‐kDa polypeptides, as well as CLN5 mutants FIN_M_, EUR, and SWE. In this study, both CLN3 and CLN5 were transfected into COS cells as this cell line did not have sufficient endogenous levels of the proteins for investigation (Vesa et al., 2002, Figure 1a).

All forms of CLN5 retained their localization to lysosomes and their ability to interact with CLN3 protein (synaptosome fraction not tested, Vesa & Peltonen, 2002). Pull‐down experiments by Lyly and colleagues in 2009 with GST‐mCLN5 also captured CLN3 protein, supporting the conclusions of Vesa and colleagues (Lyly et al., 2009). When CLN5 protein, mutated to restrict its localization to the ER, is expressed in healthy cells, it is shown to colocalize with CLN3 in the ER (Lebrun et al., 2009), the significance of this is unknown. CLN5 protein has molecular connections to CLN3 and at least to four other NCL proteins; CLN1/PPT1, CLN2/TPP1, CLN6 and CLN8 (Lyly et al., 2009). Studies using TAM‐MS combined with bioinformatics SAINT found that 18 of 31 CLN5 interactors also interacted with CLN3, further supporting the functional overlap between the two (Scifo et al., 2013).

#### 4.5.5 Interaction with CLN6

The *CLN6* gene encodes a polytopic transmembrane protein, which localizes to the ER (Heine et al., 2007, 2004; Mole et al., 2004). Mutations in the *CLN6* gene have been linked to autosomal dominant, adult‐onset known as Kuf's Type A disease. Patients with Kuf's Type A disease present with progressive myoclonic epilepsy in adulthood followed by dementia. Dissimilar to early‐onset CLN6 disease and most forms of NCL, Kuf's Type A disease does not exhibit a retinal phenotype. To test whether CLN3 and CLN6 proteins interact with one another, lymphoblast lysates were immunoprecipitated with an anti‐CLN6/CLN8 antibody and probed with a CLN3 targeted antibody. Indeed, a CLN3 band was detected, suggesting CLN6 and CLN3 physically interact. These results were confirmed by reciprocal experiments using transfected COS‐7 cells. Expression of CLN6 cDNA led to some correction of growth defects in CLN3‐deficient cells, and CLN6 was also shown to colocalize with CLN3 in fibroblasts. Together, these results suggest that CLN3 and CLN6 interact with one another (Persaud‐Sawin et al., 2007). Moreover, TAM‐MS combined with bioinformatics SAINT analysis further supports an interaction between CLN3 and CLN6; however, it is not clear whether this interaction is direct (Scifo et al., 2013).

#### 4.5.6 Interaction with CLN8

The *CLN8* gene encodes a transmembrane protein of unknown function whose ER‐Golgi intracellular location is inferred from confocal immunofluorescence microscopy of transiently transfected BHK cells (Lonka, Kyttälä, Ranta, Jalanko, & Lehesjoki, 2000). Two distinct mutations in the *CLN8* gene have been shown to result in mutation‐specific phenotypes – juvenile‐onset progressive epilepsy with mental retardation (EPMR, (Hirvasniemi, Herrala, & Leisti, 1995; Hirvasniemi & Karumo, 1994; Hirvasniemi, Lang, Lehesjoki, & Leisti, 1994) and a more severe late variant NCL with pathological similarities to CLN5‐, CLN6‐, and CLN7‐disease (Cannelli et al., 2006; Haltia, Herva, Suopanki, Baumann, & Tyynelä, 2001; Herva, Tyynelä, Hirvasniemi, Syrjäkallio‐Ylitalo, & Haltia, 2000; Ranta, Hirvasniemi, Herva, Haltia, & Lehesjoki, 2002; Ranta, Savukoski, Santavuori, & Haltia, 2001; Ranta et al., 2004; Vantaggiato et al., 2009). Expression of CLN3 cDNA in CLN8‐deficient mouse fibroblasts reduced the aberrant cellular growth of these cells. Interaction of the two proteins was observed by western blot analysis of lymphoblast lysates immunoprecipitated with an anti‐CLN8 antibody and probed with an antibody targeted at CLN3. These results were confirmed by reciprocal experiments using transfected COS‐7 cells suggesting that CLN3 and CLN8 proteins interact with one another (Persaud‐Sawin et al., 2007). Investigation of the cellular localization of CLN8 showed co‐localization of CLN3 and CLN8, which contradicts earlier studies (Persaud‐Sawin et al., 2007). However, studies using TAM‐MS combined with bioinformatics SAINT also demonstrate an interaction between CLN3 and CLN8; however, it is not clear whether this interaction is direct (Scifo et al., 2013).

#### 4.5.7 Lack of interaction with CLN4/DNAJC5, CLN7, CLN10/CTSD, CLN11/GRN, CLN12/ATP13A2/PARK9, CLN13/Cathepsin F, CLN14/KCTD7

No direct interactions between CLN3 and CLN4, CLN7, and CLN10‐14 gene products have been reported, although not all potential interactions have been explored and single‐experiment negative results may not be definitive. Moreover, it must also be considered that experimental approaches that solely interrogate direct interaction do not preclude CLN3 and the other gene products from contributing to the same cellular pathways or loosely associating in a complex.

#### 4.5.8 Lack of interaction with subunit c of mitochondrial ATP synthase; the major component of characteristic intracellular storage material build‐up

The accumulation of subunit c in CLN3 disease (Johnson et al., 1995; Westlake, Jolly, Bayliss, & Palmer, 1995) raises the possibility that CLN3 may be involved in processing or degrading subunit c, which could potentially be mediated by direct physical interaction. Using the Y2H, investigators screened fragments of the CLN3 peptide against a human fetal brain library yet failed to demonstrate a direct interaction between CLN3 and subunit c of mitochondrial ATP synthase (Leung, Greene, Munroe, & Mole, 2001).

### 4.6 Non‐bona‐fide CLN3 interactors

In addition to *bona‐fide* interactors, several other proteins have been found to bind CLN3 in vitro using the Cytotrap Y2H system. The original Y2H systems required fusion proteins to be expressed in the nucleus and were thus were not suitable for transmembrane proteins like CLN3. With Cytotrap, instead, the interaction occurs in the cytoplasm with the reporter system associated with the plasma membrane. While this technology is very useful, its use of overexpressed fusion proteins may create artificial conditions. Therefore, subsequent experiments are needed to validate findings where the only reported interaction between CLN3 and another protein of interest was as result of the use of this method.

#### 4.6.1 Interaction with myosin IIB

The C‐terminal region of CLN3 has been found to interact with myosin IIB (Getty, Benedict, & Pearce, 2011). This interaction was found by Y2H and confirmed by co‐immunoprecipitation of overexpressed CLN3 and endogenous myosin‐IIB. Non‐muscle myosin IIB interacts with adenosine triphosphate and F‐actin to promote cytoskeletal integrity and force generation for multiple cellular processes such as cell migration, shape changes, adhesion dynamics, endocytosis, exocytosis and autophagy (Heissler & Manstein, 2013). In addition to the cellular functions listed, myosin IIB has been shown to be important for numerous neuron‐specific cell functions such as polarization, dendritic spine morphology, growth‐cone motility and presynaptic vehicle trafficking. Even though the significance of the interaction between CLN3 and myosin IIB has not been fully elucidated, it stands to reason that CLN3 may act in concert with myosin IIB to regulate cytoskeletal dynamics and that the loss of CLN3 function could disrupt myosin IIB activity.

#### 4.6.2 Interaction with Shwachman‐Bodian Diamond Syndrome (SBDS) protein

To determine which proteins interact with CLN3, investigators screened fragments of the CLN3 peptide against a human fetal brain library using a cytotrap Y2H system (Vitiello et al., 2010). The C‐terminal fragment of CLN3, predicted to be cytosolic, was found to interact with the N‐terminus of Schwachman‐Bodian‐Diamond‐syndrome (SBDS) protein. These results were confirmed by co‐immunoprecipitation and co‐localization studies in NIH/3T3 cells with C‐terminal c‐myc and V5 tags (Vitiello et al., 2010). The interaction between CLN3 and SBDS is evolutionarily conserved since Sdop1 and Btn1p, the yeast homologs of SBDS and CLN3, respectively, have been found to interact with one another. Loss of SBDS protein results in Shwachman‐Bodian Diamond syndrome, an autosomal recessively‐inherited neutropenia syndrome characterized by bone marrow dysfunction and associated cumulative risk of aplastic anemia progressing to myelodysplastic syndrome and acute myeloid leukemia (Donadieu et al., 2005). Previous studies on Sdo1p revealed that this protein is involved in ribosomal biogenesis and RNA processing (Luz, Georg, Gomes, Machado‐Santelli, & Oliveira, 2009). More recently, SBDS has been found to regulate the expression of C/EBPalpha and C/EBPbeta, which are critical transcription factors for myelomonocytic lineage commitment. In particular, SBDS patients have reduced C/EBPbeta‐LIP levels (In et al., 2016). Defective expression of these factors may affect myeloid cell proliferation and differentiation, driving neutropenia which is the most prominent hallmark in almost all SBDS patients.

#### 4.6.3 Interaction with ß‐fodrin and Na+‐K+‐ATPase complex

To determine which proteins interact with CLN3 and could therefore, provide clues as to its function, investigators screened fragments of the CLN3 peptide (N1‐40 and N232‐280), both predicted to be cytoplasmic, with a LacZ/beta‐galactosidase Y2H system. Interactions were subsequently confirmed by co‐immunoprecipitation by overexpressing the full‐length CLN3 protein in COS1 cells and by using CLN3 antibodies. The results showed that full‐length CLN3 interacts with cytoskeletal protein β‐fodrin (β‐II‐spectrin) and its plasma/endosomal interaction partner Na+, K+ ATPase, a heteromeric protein with varying α‐β isoform combinations (Uusi‐Rauva et al., 2008). Beta‐fodrin, known also as non‐erythroid spectrin, is concentrated in the synaptosome fraction and is associated with synaptic membranes (Sobue, Kanda, & Kakiuchi, 1982). In erythrocyte membrane skeleton, beta fodrin has been found in heterotetrameric complexes with alpha fodrin (two alpha and two beta chains), which drive the formation of polygonal network linked to actin filaments. This network is located at the plasma membrane bilayer by interaction with ankyrin protein and the cytoplasmic domain of the Na+, K+, ATPase and it is involved in protein stability and polarization (Bennett & Baines, 2001). Na+, K+, ATPase is an ubiquitous heterodimeric transmembrane enzyme composed of varying alpha and beta isoform combinations that transport Na+ and K+ across the plasma membrane by hydrolysis of ATP (Lingrel et al., 1994). Follow‐up studies revealed that the ion pumping activity of Na+, K+ ATPase is unchanged in CLN3 disease mouse models created by a homozygous deletion of exons 1–6 of CLN3 bred onto a C57/BL genetic background. *However, the immunostaining pattern of fodrin appeared abnormal in CLN3 patient fibroblasts and Cln3^‐/‐^ mouse brains suggesting disturbances in the fodrin cytoskeleton. Furthermore, the basal subcellular distribution as well as ouabain‐induced endocytosis of neuron‐specific Na+, K+ ATPase were markedly affected in Cln3^‐/‐^ mouse primary neurons.* Studies using TAM‐MS combined with bioinformatics SAINT confirm a direct interaction between CLN3, ß‐fodrin and the Na+, K+ ATPase complex (Scifo et al., 2013; Uusi‐Rauva et al., 2008). However, the interaction between fodrin and Na+, K+, ATPase in CLN3^‐/‐^ mouse models has not been evaluated. Further studies are needed to confirm the role of CLN3 protein in the regulation of plasma membrane‐fordin cytoskeleton and, consequently, the plasma membrane association of Na+, K+ ATPase.

#### 4.6.4 Human Autophagy Interacting Network (AIN)

**Figure 11:**
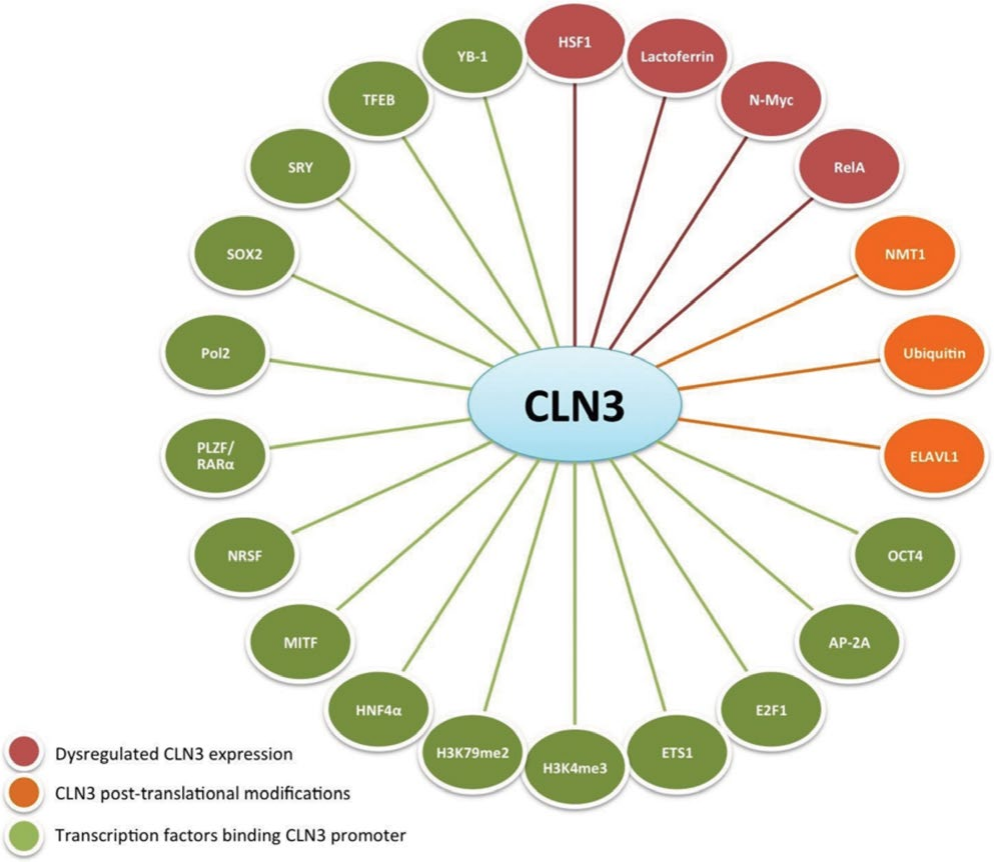
Network diagram of CLN3 interactors. These interactions have been found by ChIP‐chip and Chip‐Seq analysis (Transcription factors binding CLN3 promoter), Microarray analysis (Dysregulated CLN3 expression) and Mass spectrometry analysis (CLN3 PTMs). The majority of these interactions require further validation

However, specific aspects of the autophagy pathway have been studied extensively, less is known about the overall architecture and associated regulation of the autophagy interaction network (AIN). To generate a framework for the human AIN that could be followed up by direct mechanistic and other functional studies, Behrends and colleagues performed a systematic proteomic analysis by retrovirally expressing CLN3 and 31 additional proteins as Flag‐HA‐fusion proteins in 293T cells, isolating α‐HA immune complexes by mass spectrometry and processing the complexes using Comparative Proteomics Analysis Software Suite (*CompASS©)* to identify high‐confidence candidate interaction proteins (HCIPs). TAM‐MS and combined with bioinformatics SAINT yielded 58 CLN3 interacting partners including IMMT, GCN1L1, PRKDC, XPO1, CPT1A, HSD17B12, RPN2, PHGDH, COX15, SLC25A11, DDOST, AUP1, KIAA0368, SLC25A22, SLC25A10, and NUP205 (Scifo et al., 2013). The significance of computer‐generated results is unknown and needs to be followed up with direct mechanistic and functional studies (Figure 11).

## 5 CONCLUSION

The discovery of juvenile Batten disease occurred more than 100 years ago (Stengel, 1826). The responsible genetic dysfunction was discovered more than 20 years ago (International Batten Disease Consortium, 1995). Since that time, over 500 unique discoveries have been made relevant to the CLN3 gene, its protein, regulation or dysfunction in its absence. Despite all of this, the function of CLN3 protein remains elusive. The authors hope that the historical research findings presented here will contribute to determining the function of the CLN3 protein and exactly why, CLN3 gene defects adversely affect endosomal‐lysosomal homeostasis, proteome, lipidome, trafficking, maturation, and recycling activities (anHaack et al., 2011; Appu et al., 2019; Burkovetskaya et al., 2014; Cao et al., 2006; Chandrachud et al., 2015; Codlin et al., 2008; Codlin & Mole, 2009; Gachet et al., 2005; Golabek et al., 2000; Holopainen, Saarikoski, Kinnunen, & Jarvela, 2001; Fossale et al., 2004; Kama et al., 2011; Luiro et al., 2004; Metcalf, Calvi, Seaman, Mitchison, & Cutler, 2008; Schmidtke et al., 2019; Stein et al., 2010; Tecedor et al., 2013; Vidal‐Donet, Cárcel‐Trullols, Casanova, Aguado, & Knecht, 2013). Why does CLN3 protein deficiency impact protein palmitoylation? How then does palmitoylated reversible trafficking and localization of proteins to cell membranes contribute to cell death (Narayan et al., 2006, 2008)? Are the defects found in the endoplasmic reticulum, trans‐golgi network, and mitochondria of CLN3‐deficient cells primary or secondary to disease pathogenesis (Fossale et al., 2004; Luiro et al., 2006; Metcalf et al., 2008)? If the breakdown in intracellular communication between membranous compartments of the endosomal‐lysosomal system and other organelles were resolved in glia, would neuronal survivability improve (Parviainen et al, 2017)? Which defects lead to the selective vulnerability of neurons in disease? Overexpression of CLN3 protein has been reported in several primary cancerous tissues and cell lines (Dhar et al., 2002; Makoukji et al., 2015; Mao et al., 2015; Narayan et al., 2006; Oltulu et al., 2019; Ren et al., 2016; Rylova et al., 2002; Xu et al., 2019; Zhang et al., 2008; Zhu et al., 2014). Are the anti‐apoptotic properties of CLN3 protein responsible for promising preclinical CLN3 gene therapy reports or does gene therapy affect one or more of the deficient processes listed above (Bosch et al., 2016; Lane, Jolly, Schmechel, Alroy, & Boustany, 1996).

## CONFLICT OF INTEREST

The authors certify that they have NO affiliations or involvement in any organization or entity with any financial interest (such as honoraria; educational grants; participation in speakers’ bureaus; membership; employment; consultancies; stock ownership, or other equity interest; and expert testimony or patent licensing arrangements), or nonfinancial interest (such as personal or professional relationships, affiliations, knowledge or beliefs) in the subject matter or materials discussed in this manuscript.

## APPENDIX 1

### ABBREVIATIONS

1. aa: amino acid
2. AIN: (Human) Autophagy Interacting Network
3. ATP: Adenosine triphosphate
4. BHK: baby hamster kidney
5. bp: base pair
6. CLN1: ceroid‐lipofuscinosis, neuronal 1
7. CLN2: ceroid‐lipofuscinosis, neuronal 2
8. CLN3: ceroid‐lipofuscinosis, neuronal 3
9. CLN4: ceroid‐lipofuscinosis, neuronal 4
10. CLN5: ceroid‐lipofuscinosis, neuronal 5
11. CLN6: ceroid‐lipofuscinosis, neuronal 6
12. CLN7: ceroid‐lipofuscinosis, neuronal 7
13. CLN8: ceroid‐lipofuscinosis, neuronal 8
14. DREAM: downstream regulatory element antagonist modulator
15. EEA1: early endosome antigen 1
16. EPMR: Progressive Epilepsy with Mental Retardation
17. GFP: Green Flourescent Protein
18. GST: Glutathione S Transferase
19. GTP: guanosine triphosphate
20. hp: haptoglobin
21. INCL: infantile neuronal ceroid lipofuscinosis
22. JNCL: juvenile Neuronal Ceroid Lipofuscinosis
23. kb: kilobase
24. kDa: kilodalton
25. LINCL: late infantile Neuronal Ceroid Lipofuscinosis
26. M6P: mannose 6 phosphate
27. MFS: Major Facilitator Superfamily
28. MSD: Membrane Spanning Domain
29. NCBI: National Center for Biotechnology Information
30. NLM: National Library of Medicine
31. NRK: Normal Rat Kidney
32. PDI: protein disulfide isomerase
33. PKA: protein kinase A
34. PKG: protein kinase G
35. PPT1: palmitoyl‐protein thioesterase 1
36. PTM: post‐translational modification
37. RFU: relative fluorescent units
38. S. cerevisiae: schizosaccharomyces cerevisae
39. S. pombe: schizosaccharomyces pombe
40. SBDS: Shwachman‐Bodian Diamond Syndrome
41. SNP: Single Nucleotide Polymorphism
42. TAM‐MS: Tandem Affinity Purification coupled with Mass Spectrometry
43. TPP1: Tripeptidyl Peptidase 1
44. TSS: Transcription Start Site
45. UTR: Untranslated Region
46. vLINCL fin: Finnish variant late infantile form of Neuronal Ceroid Lipofuscinosis
47. Y2H: yeast‐2‐hybrid
