# Supplementary material for: The CLN3 gene and protein: What we know
Source: Mol Genet Genomic Med. 2019 Sep 30;7(12):e859. doi: 10.1002/mgg3.859 (PMC6900386; doi:10.1002/mgg3.859)
Supplement: Supplementary file 1 [file MGG3-7-e859-s001.docx]

CLN3 Research Tools and Resources

*Original References*

**Amoeba**

1. **Dictyostelium discoideum**
   - Huber RJ, Myre MA, Cotman SL. Loss of CLN3 function in the social amoeba Dictyostelium discoideum causes pleiotropic effects that are rescued by human CLN3. (Huber, Myre, and Cotman 2014)

**Yeast Models**

1. **Schizosaccharomyces cerevisae**
   - Pearce DA, Sherman F. A yeast model for the study of Batten disease. (Pearce and Sherman 1998)
2. **Schizosaccharomyces pombe**
   - Gachet Y, Codlin S, Hyams JS, et al. BTN1, the Schizosaccharomyces pombe homologue of the human Batten disease gene CLN3, regulates vacuole homeostasis. (Gachet et al. 2005)

**Roundworm**

1. **Caenorhabditis elegans**
   - Voer G1, van der Bent P, Rodrigues AJ, et al. Deletion of the Caenorhabditis elegans homologues of the CLN3 gene, involved in human juvenile neuronal ceroid lipofuscinosis, causes a mild progeric phenotype. (de Voer et al. 2005)

**Fruitfly**

1. **Drosophila melanogaster - Genetic gain-of-function system**

- Tuxworth RI, Chen H, Vivancos V, et al. The Batten disease gene CLN3 is required for the response to oxidative stress. (Tuxworth et al. 2011)

**Zebrafish**

1. **Danio rerio - Morphant**

- Wager K, Zdebik AA, Fu S, et al. Neurodegeneration and Epilepsy in a Zebrafish model of CLN3 Disease (Batten Disease). (Wager et al. 2016)

**Mouse Models**

1. **Mus musculus - Deletion of exons 2-6 and most of exon 1 on 129/Sv inbred and 129Sv3 Black Swiss outbred lines**

- Mitchison HM, Bernard DJ, Greene ND, et al. Targeted disruption of the CLN3 gene provides a mouse model for Batten disease. (Mitchison et al. 1999)

1. **Mus musculus - Cln3Δex7/8 knock-in mice bearing the common** ∼**1 kb deletion on an outbred 129Sv-CD1 background**

- Cotman SL, Vrbanac V, Lebel LA, et al. [Cln3(Deltaex7/8) knock-in mice with the common JNCL mutation exhibit progressive neurologic disease that begins before birth.](http://www.ncbi.nlm.nih.gov.ezproxy.galter.northwestern.edu/pubmed/12374761) (Cotman et al. 2002)

1. **Mus musculus - Targeted replacement of most of exon1 and all of exons 2-8 of the CLN3 gene with β-galactosidase on SV129 and onto C57BL/6**

- Eliason SL, Stein CS, Mao Q, et al. A knock-in reporter model of Batten disease. (Eliason et al. 2007)

1. **Mus musculus - Cln3Δex7/8 mice on a C57BL/6J background with a light-sensitive Rpe65 leucine variant**

- Dannhausen K, Möhle C, Langmann T. Immunomodulation with minocycline rescues retinal degeneration in juvenile neuronal ceroid lipofuscinosis mice highly susceptible to light damage. (Dannhausen et al. 2018).

**Animal Cell lines**

1. **Mus musculus - Homozygous CbCln3Deltaex7/8 precursor cells**

- Fossale E, Wolf P, Espinola JA, et al. Membrane trafficking and mitochondrial abnormalities precede subunit c deposition in a cerebellar cell model of juvenile neuronal ceroid lipofuscinosis.

1. **Mus musculus - Mouse Brain Endothelial Cells**

- Tecedor L, Stein CS, Schultz ML, et al. CLN3 Loss Disturbs Membrane Microdomain Properties and Protein Transport in Brain Endothelial Cells. (Tecedor et al. 2013)

**Human**

1. **CLN3 deficient**
   - **Homo sapien - Human CLN3 Knockout cell line 19 bp deletion**
     - - <https://www.horizondiscovery.com/human-cln3-knockout-cell-line-hzghc003369c002>
     - **Homo sapien - Human CLN3 Knockout cell line 8 bp deletion**
       - <https://www.horizondiscovery.com/human-cln3-knockout-cell-line-hzghc003369c010>
     - **Panoply™ Human CLN3 Knockdown Stable Cell Line(CSC-DC003300)**
       - <http://www.creative-biogene.com/Panoply-Human-CLN3-Knockdown-Stable-Cell-Line-CSC-DC003300-1256319-14.html>

- **Homo sapien - Induced pluripotent stem cells**
  - - - Lojewski X, Staropoli JF, Biswas-Legrand S, et al. Human iPSC models of neuronal ceroid lipofuscinosis capture distinct effects of TPP1 and CLN3 mutations on the endocytic pathway. (Lojewski et al. 2014)
      - New York Stem Cell Foundation scientists created a collection of iPS cells from skin samples and the NYSCF Global Stem Cell Array™, an automated robotic technology that standardizes and scales stem cell production and differentiation, enabling the manufacture and analysis of large numbers of identical cells further described in [www.ncbi.nlm.nih.gov/pubmed/26237226](http://www.ncbi.nlm.nih.gov/pubmed/26237226). For a list of stem cell lines available and information on acquiring the cell lines, visit [www.nyscf.org/repository](http://www.nyscf.org/repository). Additional lines are in development, including isogenic controls of currently available lines and genetically-engineered mutants. These will be made available on an ongoing basis when completed.

1. **CLN3 overexpression**

- **Homo sapien - Cancer cell lines**
- Rylova SN, Amalfitano A, Persaud-Sawin DA, et al. The CLN3 gene is a novel molecular target for cancer drug discovery. (Rylova et al. 2002)
- Glioblastoma (U-373G, T98g)
- Neuroblastoma (IMR-32, SH-SY5Y, SK-N-MC)
- Prostate (Du145, PC-3, LNCaP)
- Ovarian (SK-OV-3, SW626, PA-1)
- Breast (BT-20, BT-549, BT-474)
- Zhu X, Huang Z, Chen Y, et al. Effect of CLN3 silencing by RNA interference on the proliferation and apoptosis of human colorectal cancer cells. (Zhu et al. 2014)
- Colon (SW1116, SW480, HCT 116)
- **Panoply™ Human CLN3 Knockdown Stable Cell Line(CSC-DC003300)**
  - <http://www.creative-biogene.com/Panoply-Human-CLN3-Over-expressing-Stable-Cell-Line-CSC-SC003300-1237907-15.html>

**NCL Mutation and Patient Database** <http://www.ucl.ac.uk/ncl/mutation.shtml>

“This database contains published mutations and sequence variations in genes that cause NCL together with unpublished data included with permission. It follows the mutation nomenclature recommendations of the Human Genome Variation Society. From mid-2012, there are now two sets of tables for each human NCL disease gene - the new Patient Database listing all published or reported patients and families, and the Mutation Database listing all published or reported mutations and many sequence variants as before, and now cross-referenced to the patient table. These are available to view via this web site and also to download as excel files for off-site use to aid local needs or interests (e.g. sorting according to occurrence in particular countries).”
